# Effect of Embossing Pattern Geometry on the Tensile Strength of Three-Layer Films Containing Recycled Polyethylene

**DOI:** 10.3390/ma19214657

**Published:** 2026-11-01

**Authors:** Jachowicz Tomasz, Woźniak Waldemar, Sąsiadek Michał, Niedziela Maciej, Jajczyk Jarosław, Arkadiusz Kozioł, Tomasz Tramś

**Affiliations:** 1 Faculty of Mechanical Engineering, Lublin University of Technology, 38 Nadbystrzycka Str., 20-618 Lublin, Poland; 2 Institute of Materials and Biomedical Engineering, University of Zielona Góra, 4 Prof. Z. Szafran Str., 65-516 Zielona Góra, Poland; w.wozniak@iimb.uz.zgora.pl; 3 Institute of Mechanical Engineering, University of Zielona Góra, 4 Prof. Z. Szafran Str., 65-516 Zielona Góra, Poland; m.sasiadek@iim.uz.zgora.pl; 4 Institute of Mathematics, University of Zielona Góra, 4 Prof. Z. Szafran Str., 65-516 Zielona Góra, Poland; m.niedziela@wmie.uz.zgora.pl (N.M.); a.koziol@wmie.uz.zgora.pl (A.K.); 5 Faculty of Control, Robotics and Electrical Engineering, Poznań University of Technology, 3A Piotrowo Str., 60-965 Poznań, Poland; jaroslaw.jajczyk@put.poznan.pl; 6 Polipak sp. z o.o., Fabryczna 7, 63-000 Środa Wielkopolska, Poland; t.trams@polipak.com.pl

**Keywords:** polyethylene multilayer films, embossing, recycled polyethylene content, mechanical properties

## Abstract

This paper presents the results of experimental tests on the static tensile strength and relative elongation of three-layer films containing varying proportions of recycled polyethylene and subjected to an embossing process. Films containing varying proportions of recycled low-density and high-density polyethylene were produced by blown film extrusion. The embossing process was carried out under varying processing conditions, and embossing patterns with different geometrical characteristics were produced on the three-layer films under investigation. The films were manufactured and the experimental tests were conducted under industrial conditions. Based on the results obtained, the effects of three different embossing pattern geometries and the embossing process parameters on the tensile strength of three-layer films made from recycled polyethylene were evaluated in the machine and transverse directions. The experimental results enabled the identification of embossing pattern geometries and recycled polyethylene contents that increased static tensile strength in selected material–process combinations. No statistically significant improvement in elongation at break was confirmed compared with the non-embossed reference films. The findings are limited to static tensile strength and elongation at break in the machine direction (MD) and cross direction (CD), which contribute to improving certain functional properties of refuse bags, specifically ensuring adequate load-bearing capacity for the waste they contain.

## 1. Introduction

Blown film extrusion is one of the most important manufacturing technologies used for the continuous production of thin tubular films. This process, which is used for thermoplastic polymers, is based on the simultaneous stretching of the material in two directions: axially, by means of a take-up system, and circumferentially, by means of air pressure inside the tubular film. The process produces a tubular film with a high degree of molecular orientation, resulting in favorable mechanical properties [1,2].

The blown film extrusion process is carried out using a specially designed extrusion line. First, the polymer granules are plasticised and thermally homogenised in a screw extruder. The polymer melt is then conveyed under high pressure to an extrusion die equipped with an annular opening. Upon leaving the die, the molten polymer forms a tube into which compressed air is introduced. This causes the tubular film to expand to the desired diameter. A key process parameter is the blow-up ratio, defined as the ratio of the diameter of the inflated film tube to the diameter of the extrusion die opening. The film tube is cooled externally by a cooling ring that directs an air stream onto the film surface.

The point at which the polymer transitions from the molten state to the solid state is referred to as the frost line [3,4]. The stabilised film tube then passes through a calibration cage and a collapsing unit, after which it is drawn off by a set of nip rolls and finally wound into rolls. Several variants of this method can be distinguished: vertically upward extrusion, which is the most common, downward vertical extrusion and horizontal extrusion [5,6,7].

Today, the free-blown film extrusion process is used to produce single- and multilayer films from a wide range of polymers, with a broad range of thicknesses and exhibiting diverse performance characteristics, such as high barrier properties. The main advantages of this technology include the ability to produce films of very large widths—up to several metres after slitting; the absence of edge-trim waste, unlike in cast-film extrusion; high mechanical strength resulting from biaxial polymer orientation; and easy adjustment of the film thickness and width without the need to replace the die. The disadvantages include lower cooling efficiency compared with cast-film extrusion which limits the film line speed, reduced film transparency resulting from slower crystallisation, and the sensitivity of the process to external conditions, including air turbulence in the production hall, which may lead to variations in film thickness [8,9].

Embossed film is a material that has undergone a mechanical process in which a geometric pattern is impressed onto its surface using specially engraved rollers. Although it may appear to be merely an aesthetic treatment, the permanent pattern produced by mechanical embossing serves several important technical and functional purposes, particularly in refuse bag production. Embossing imparts a textured surface to the film, thereby modifying selected functional properties, including preventing blocking between film layers and improving optical properties by effectively concealing the bag’s contents. From a processing perspective, embossed film also improves process stability by reducing friction against metal machine components [10,11,12].

Multilayer films are produced by coextrusion, in which several streams of polymer melt are simultaneously fed through a common extrusion die. The polymer material for each layer is supplied by a separate extruder, allowing precise control of the thickness and composition of the individual layers within the film cross-section. The coextrusion of multilayer films containing recycled material is currently one of the most important trends in plastics processing, driven by the transition towards a circular economy [13,14,15]. This technology makes it possible to combine the advantages of different polymers and to use recycled raw materials efficiently without significantly compromising the quality of the final product.

When recycled polymers are used, three- or five-layer structures are most commonly employed. In a three-layer structure, the outer layers are made from virgin polymer and provide the required optical properties, including gloss, as well as sealability and functional barrier properties, while the middle layer forms a core containing the recycled material. The core layer may account for between 30% and 70% of the total film thickness. Incorporating recycled material into the inner layer helps to conceal its imperfections, as recycled polymers often contain residual pigments, mechanical inclusions, or a characteristic odour. Encapsulating these materials between layers of virgin polymer prevents the recycled material from coming into direct contact with the packaged product or the external environment.

The key technological considerations associated with the efficient production of multilayer films containing recycled material include: (a) proper filtration of the recycled feedstock, requiring the use of advanced melt-filtration systems in the extruder, such as automatic screen changers fitted with fine-mesh screens; (b) compatibilisation, which is necessary when the recycled material consists of a blend of different polymers and may therefore require the use of tie layers or compatibilising agents to prevent film delamination; and (c) homogenisation, requiring specially designed screw geometries in the extruder plasticising systems to ensure stable pressure in the extrusion die when the recycled feedstocks used exhibit variable melt flow rates.

The principal advantages of incorporating recycled polymers into multilayer films include compliance with environmental and legislative requirements through a reduced carbon footprint and the fulfilment of recycled-content targets for packaging; lower production costs, as recycled feedstock is generally less expensive than virgin material; and improved stability of the blown film tube due to the presence of cross-linked polymer fractions in recycled materials, which may enhance the mechanical strength of the film. The disadvantages include deterioration of the film’s optical properties, such as increased haze; the risk of contaminant migration due to the reduced barrier performance of recycled polymers; and a higher incidence of disruptions in the blown film extrusion process caused by screen clogging and the need for more frequent replacement of the filtration screens [16,17,18,19].

## 2. Subject of the Study

The materials investigated comprised six selected formulas of three-layer polyethylene film produced by blown film extrusion. Two nominal recycled-polyethylene contents, 54% and 66%, were investigated, corresponding to calculated total mass fractions of 54.2% and 65.8%, respectively (Table 1). These fractions include both recycled LDPE and recycled HDPE; the remaining constituents comprise virgin polyethylene and additives. The labels 54LDPE, 54HDPE, and 66HDPE identify material groups and nominal recycled-content levels rather than independently controlled, exact local concentrations in the film.

The industrial trials covered a broader set of film formulations, of which six met the predefined target values for the investigated static tensile-strength and relative-elongation indicators under the industrial processing conditions used. These six formulations were therefore selected for the present analysis. The industrial production setting nevertheless restricted systematic sampling at additional recycled-content levels. Variability in recycled-feedstock properties, dosing, and material flow through the extrusion die complicates the maintenance of constant local composition and layer proportions. Accordingly, the calculated values and nominal group labels should not be interpreted as proof of identical composition throughout the film. Potential systematic deviations associated with dosing and die flow were not quantified in the present analysis; no numerical composition uncertainty is therefore assigned. The results and conclusions of the present analysis are limited to the six selected formulations and the processing conditions used; they do not establish a continuous processing window above 54% recycled content.

The recycled-polyethylene content was calculated from the gravimetrically determined material consumption for all three layers as the total mass of recycled LDPE and HDPE supplied to the three layers divided by the total mass of processing material, including virgin polyethylene and additives, multiplied by 100%. Thus, the reported contents are average, mass-balance-based values for the complete three-layer film, rather than direct measurements of the recycled fraction at each sampling position. The outer and inner layers served as protective skins around the middle layer, into which the more variable recycled feedstock was introduced.

Three-layer films with an average thickness of 24–27 μm were subjected to permanent mechanical embossing using an embossing unit equipped with two working rollers. The embossing unit enabled adjustment of the film line speed and the embossing roller axis distance. Three embossing patterns with different geometric characteristics, designated E1, E2, and E3, were produced using three separate sets of working rollers. Formulas E2 and E3 were selected for basic experimental studies and statistical evaluation.

**Figure 1:**
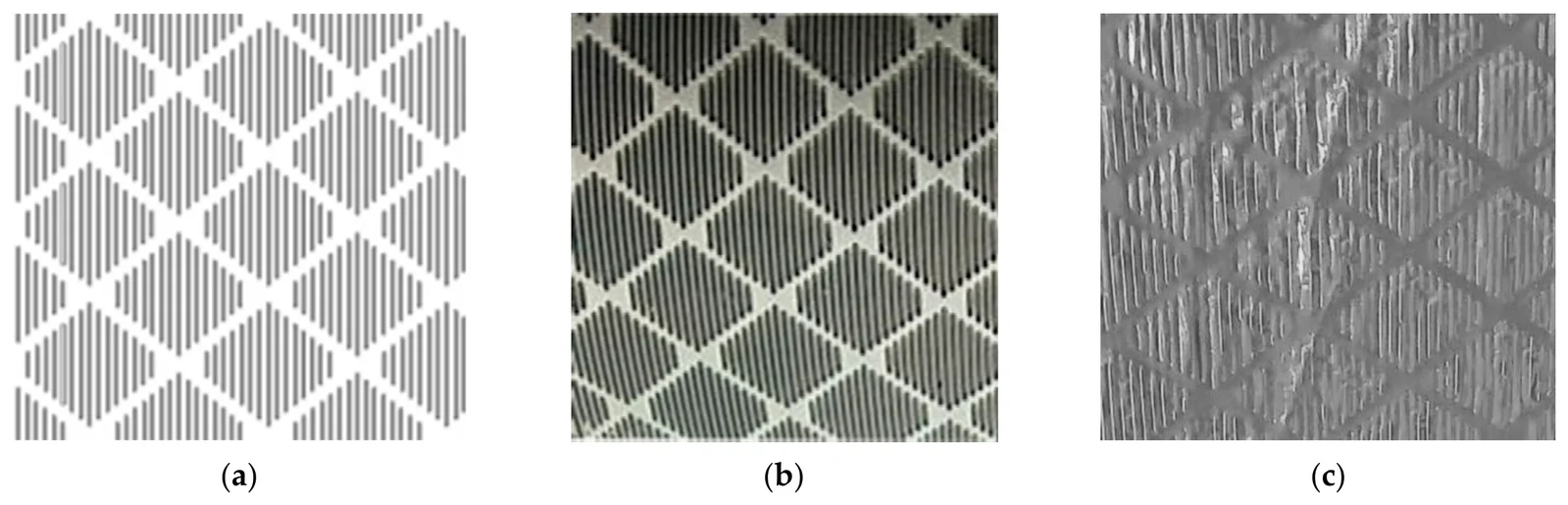
Embossing pattern E1: (**a**) geometry of the embossed pattern, (**b**) photograph of a section of the embossing roller surface, and (**c**) photograph of a section of the embossed three-layer film surface.

**Figure 2:**
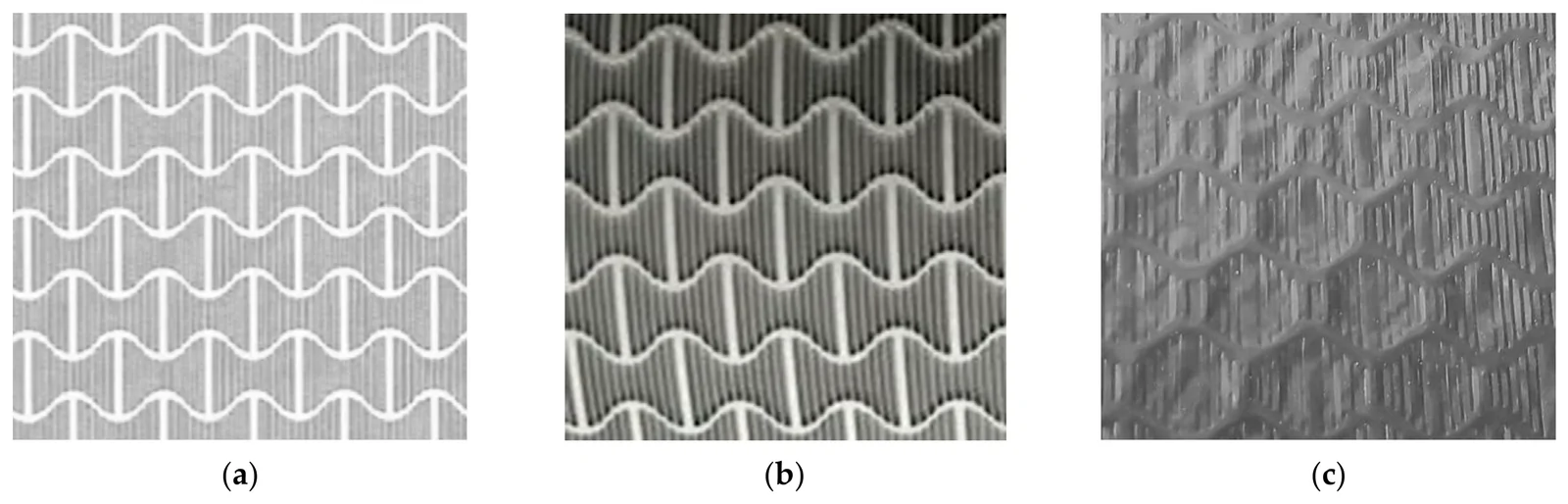
Embossing pattern E2: (**a**) geometry of the embossed pattern, (**b**) photograph of a section of the embossing roller surface, and (**c**) photograph of a section of the embossed three-layer film surface.

**Figure 3:**
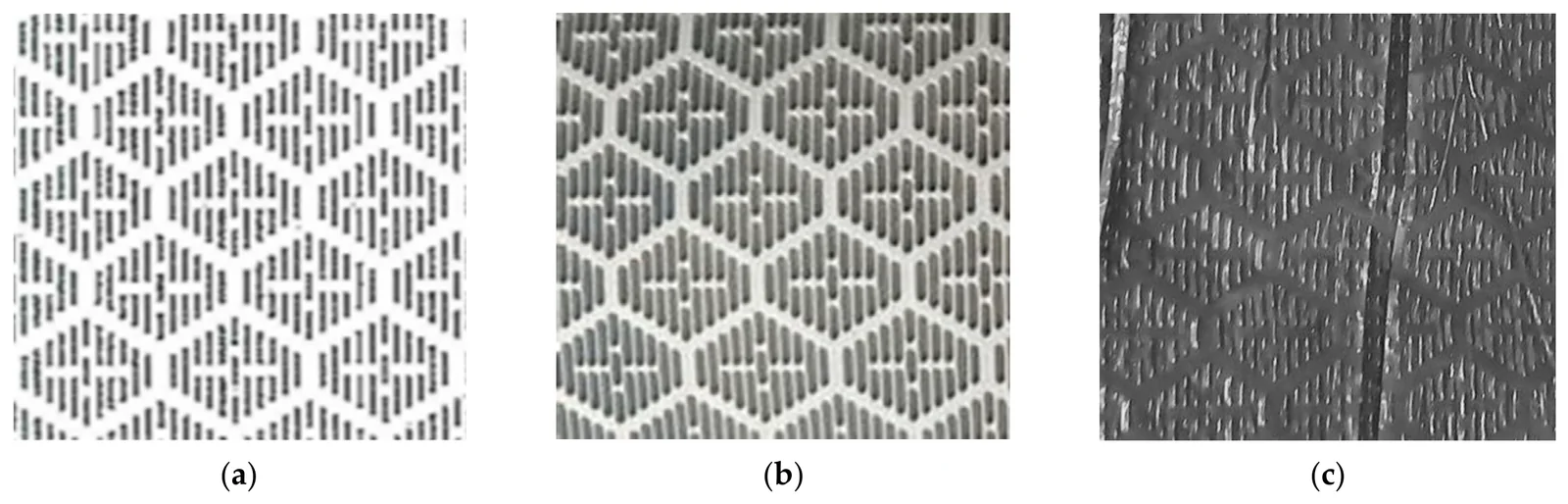
Embossing pattern E3: (**a**) geometry of the embossed pattern, (**b**) photograph of a section of the embossing roller surface, and (**c**) photograph of a section of the embossed three-layer film surface.

The geometries of the embossing patterns, the surfaces of the embossing rollers and the embossed films are shown in Figure 1, Figure 2, and Figure 3, respectively. For all three patterns, the grooves forming the embossing pattern on the embossing rollers were 1.5 mm deep.

## 3. Aim and Scope of the Study

The aim of the study was to determine the effects of embossing pattern geometry and embossing process on the static tensile strength and relative elongation of three-layer polyethylene films with varying recycled polyethylene content. Measurements were performed in the machine and cross directions. The scope is limited to these two responses under the static tensile-test conditions used.

The variable factors in the production of embossed three-layer film containing recycled polyethylene were the embossing pattern geometry, the centre-to-centre distance between the embossing rollers, and the line speed of the three-layer film.

The three embossing pattern geometries, designated E1, E2, and E3, were defined by the shapes and dimensions of the elements forming the embossing profile, as shown in Figure 1, Figure 2 and Figure 3. Following the preliminary tests, two of the three embossing patterns, designated E2 and E3, were selected for further testing under industrial conditions.

The centre-to-centre distance between the embossing rollers, hereinafter referred to as the roller axis distance (RAD), controlled the embossment depth. In the industrial trials, a ten-level adjustment scale for the RAD (ranging from 0 to 9, was used. The RAD was adjusted using a graduated screw mechanism. Setting the adjustment screws to 0 corresponded to the minimum RAD, with the embossing elements engaging to the maximum depth permitted by their geometry and dimensions. Increasing the distance between the roller axes resulted in a gradual increase in the gap between the rollers. When the adjustment screws were set to position 9, the roller axes were at their maximum separation, and consequently the embossing elements did not engage with one another. The method used to set the RAD and the resulting graduated adjustment of the pressure applied to the three-layer film during embossing form part of the company’s proprietary design know-how. Therefore, the corresponding absolute geometric values cannot be disclosed in this article.

The line speed of the three-layer film was determined by the design of the processing line and was set at 150, 165, 180, and 195 m/min.

## 4. Methodology of Experimental Research

The static tensile properties of the three-layer films were determined in accordance with PN-EN ISO 527-1:2020 and PN-EN ISO 527-3:2019. A Testometric X250-2.5 universal testing machine from Testometric Ltd. (Rochdale, UK) was used to perform the tests. The testing machine was connected to a PC. WinTest Analysis EC software (v.6.2) was used to control the tests and to acquire and process the measurement data.

The test specimens were rectangular and measured 15 × 150 mm. The use of rectangular specimens is permitted under PN-EN ISO 527-3:2019. The specimens were cut mechanically using a cutting die and prepared to ensure that they were free from surface and material defects. They were cut parallel and perpendicular to the axis of the extruded tubular film. Seven film specimens were typically prepared for each test series so that, after the possible exclusion of two clearly outlying results—the lowest and the highest—at least five valid specimens remained.

The industrial-scale study covered six three-layer film formulations: F004, F032, F045, F046, F047, and F050. For each formulation, three production trials were conducted using distinct production batches, with two rolls of three-layer film obtained from each trial. For each tested combination of formulation, embossing pattern, embossing roller axis distance (RAD), and line speed, seven test specimens were taken from each roll to measure tensile strength and elongation at break in the machine direction (MD) and cross direction (CD).

The specimens were conditioned for at least 24 h at a temperature of 23 ± 2 °C and a relative humidity of 50 ± 10%. The specimen extension rate was constant and was set at 100 mm/min for the films tested. WinTest Analysis EC software enabled the required relationships, such as stress–strain curves, to be visualised directly on the monitor and the relevant mechanical properties to be calculated. The software also enabled both the measurement data and the calculated results to be exported in tabular and graphical form.

## 5. Statistical Analysis Methods

Statistical analysis of the experimental results was performed to determine whether embossing and variations in the embossing process parameters had a significant effect on the static tensile properties of the three-layer films. The analysis was conducted separately for specimens cut in the machine direction (MD), parallel to the direction of film extrusion, and in the cross direction (CD). Static tensile strength σ_r_ and relative elongation ε_r_ were used as the response variables. The comparative analyses also included comparisons with the results obtained for non-embossed films with corresponding material characteristics.

In the second stage, only the results obtained for the embossed films were compared in order to determine the effects of the individual material and processing factors on tensile strength and relative elongation in the MD and CD. This part of the analysis considered the material group, embossing pattern, roller axis distance, and film line speed. A similar approach, involving the comparison of product properties obtained at different processing parameter settings, has also been adopted in other experimental studies of manufacturing processes and production parameters [20,21].

Before selecting the appropriate statistical tests, a preliminary diagnostic assessment of distributional properties was performed using the Shapiro–Wilk test. While the Shapiro–Wilk test provided initial diagnostic insights, non-parametric statistical methods were chosen as the principal analytical framework primarily due to their robust operational properties. Non-parametric procedures are substantially more resilient to severe distributional skewness, non-constant variance, unequal sample sizes across experimental design cells, and physical outliers inherent to data generated during industrial pilot trials.

To control the family-wise error rate arising from performing multiple one-sample tests across various factor combinations, raw *p*-values were adjusted using Holm’s step-down procedure (Holm–Bonferroni correction). Furthermore, to evaluate the practical and technological magnitude of property improvements beyond mere statistical significance, non-parametric effect sizes (rank biserial correlation coefficient for Wilcoxon tests and dominance statistics for sign tests). Effect sizes provide a sample-size-independent metric quantifying the strength of the embossing effect, categorized as large, when effect size is greater than 0.6.

To compare the results obtained for the embossed films with a predefined reference value, two one-sample non-parametric tests were used: the Wilcoxon signed-rank test and the sign test. The Wilcoxon test is used, among other applications, in statistical procedures for verifying measurement results and technical parameters [22,23]. The sign test, by contrast, is a distribution-free method that uses only information on the direction of the difference between an observation and the reference value, making it robust to pronounced distributional asymmetry and the presence of outliers [24].

For each factor combination, one-sided tests were performed to determine whether the tensile-property values for embossed films were shifted above the corresponding reference value. The Wilcoxon signed-rank test assessed a positive location shift under the assumption of approximate symmetry of the differences, whereas the sign test assessed whether the median difference was greater than zero without requiring distributional symmetry. The minimum tensile strength recorded for the corresponding non-embossed film was adopted as a conservative reference baseline. This lower-bound benchmark was chosen to evaluate whether embossing consistently shifts product strength above the minimum threshold of non-embossed films. As a methodological limitation, one-sample tests against this minimum value demonstrate that embossed films exceed this baseline threshold, but do not imply superiority over the entire distribution of non-embossed films. The Wilcoxon signed-rank test was used as the primary method for assessing shifts in the distribution of the results, whereas the sign test was used as a complementary, more robust method for evaluating the direction of change, particularly for smaller samples or in the presence of a greater number of outliers.

Non-parametric multifactorial methods were used to assess the effects of the embossing process parameters on the tensile strength of the embossed films. The Scheirer–Ray–Hare test was applied as an initial screening tool. This test is a rank-based extension of the Kruskal–Wallis test for the experimental designs involving more than one grouping factor [25]. This test made it possible to assess whether the main grouping factors, such as the embossing pattern, recycled polyethylene content and type, embossing roller axis distance, and film line speed, had a significant effect on tensile strength. In addition, potential interactions between the factors were analysed, as the effect of the embossing pattern may depend on the material composition of the film or the process settings. However, recognizing its relatively low statistical power for detecting multi-factor interaction effects, the analysis was supplemented by Aligned Rank Transform Analysis of Variance (ART ANOVA). ART ANOVA provides enhanced sensitivity for non-parametric evaluation of main and interaction effects in unbalanced factorial designs. All higher-order interaction analyses were treated as exploratory in nature. A non-significant interaction result in these tests was interpreted as a lack of sufficient sample evidence to confirm an interaction, rather than definitive proof that no joint factor effect exists.

Because of the limited ability of the Scheirer–Ray–Hare test to detect more complex interactions, the analysis was supplemented by aligned rank transform analysis of variance (ART ANOVA). Methods based on aligned rank transformations were developed to enable the non-parametric analysis of main and interaction effects in multifactorial experiments [26,27]. The procedure first involves isolating the effect under investigation by aligning the data and then replacing the aligned values with their ranks. A conventional analysis of variance to test the effect of interest is then performed on the transformed data.

The use of the aligned rank transform enabled higher-order interaction effects to be assessed without requiring the original data to be normally distributed. The results of this analysis were interpreted as complementary to those obtained using the Scheirer–Ray–Hare test. A main effect or interaction effect was considered statistically significant only when the corresponding *p*-value was lower than the adopted significance level.

Because experimental trials were conducted under industrial pilot conditions, extreme parameter combinations (e.g., maximum line speed coupled with maximum embossing depth) occasionally caused film rupture or process instability, resulting in an unbalanced design matrix with unpopulated factor combinations. To address this design structure without introducing estimation bias, Aligned Rank Transform Analysis of Variance (ART ANOVA) was conducted using Type III Sums of Squares. Type III Sums of Squares test each effect marginally after adjusting for all other main effects and interaction terms, thereby preventing unequal cell frequencies from biasing lower-order main effect estimates. Sparse factor combinations with *n* < 3 observations were excluded prior to model fitting to maintain estimation stability.

When the overall analysis indicated a statistically significant effect of a factor, post hoc pair-wise comparisons were performed using Dunn’s test. This test was used to identify which specific levels of the factor analysed differed significantly from one another and, hence, which material groups, embossing patterns, roller axis distance settings, or film line speeds accounted for the differences detected by the overall test. Because multiple pairwise comparisons were performed simultaneously, the *p*-values were adjusted to account for multiple testing. This reduced the risk of a Type I error, in which a difference arising by chance is incorrectly considered statistically significant.

A significance level of α = 0.05 was adopted for all analyses. A result was considered statistically significant when the adjusted *p*-value was less than 0.05. The results of the analyses are presented later in the article in the form of box plots and tables reporting the *p*-values and the sample sizes for the groups analysed.

Box plots were used to illustrate the distributions of the results for the individual combinations of factors, including the material group, embossing pattern, and roller axis distance setting. In addition to the spread of the results, the position of the median, and the presence of outliers, these plots also indicated the reference value corresponding to the minimum tensile strength of the non-embossed film for the corresponding material group. This value served as the reference level in the statistical tests used to determine whether embossing resulted in a significant increase in tensile strength. The statistical test tables, in turn, were used to identify the material–process combinations for which the observed differences in tensile strength were statistically significant.

## 6. Experimental Results

Given the wide range of levels of the factors investigated and the resulting combinations used in the production of the three-layer films from which the test specimens were subsequently prepared, it was not possible to present every individual measurement result within the scope of this scientific article. This section therefore presents representative measurement results and summaries for the individual measurement groups, primarily in the form of mean values, whereas the statistical analysis, which was based on all individual measurements, is presented later in the article.

Since the tests were conducted under industrial conditions, it was not possible to perform identical trials for films produced using all formulas. With some combinations of processing parameters, the films either ruptured, the process became unstable, or the resulting embossment failed to meet the aesthetic requirements. Consequently, the measurement groups for the individual formulas differed, particularly in terms of the ranges of embossing roller axis distance settings and film line speeds, as the principal criterion during testing was to obtain a correctly formed embossed pattern while maintaining stable processing conditions.

Table 2 presents the mean tensile strength in the machine and cross directions, together with the corresponding relative elongation, of non-embossed three-layer films produced according to the selected formulas. The reported results were used as reference data for comparison with those obtained for the embossed films.

Based on the type and total content of waste polymer, the formulas were divided into three groups: 54HDPE, comprising formulas F046 and F047; 54LDPE, comprising formulas F004 and F032; and 66HDPE, comprising formulas F045 and F050, which contained a mixture of LDPE and HDPE with a clear predominance of HDPE.

Table 3 presents an example of a partial report summarising the mean values of static tensile strength and relative elongation determined for a group of specimens prepared from a three-layer film produced according to a single formula and embossed with one embossing pattern at different film line speeds and embossing roller axis distance settings. Twelve such partial reports were prepared and analysed, covering all the formulas and embossing patterns investigated.

## 7. Statistical Analysis of the Experimental Results

Based on the experimental results, the statistical analysis was performed separately for the machine direction (MD) and cross direction (CD), taking into account the mean values of tensile strength, σ_r_, and relative elongation, ε_r_, expressed relative to the results obtained for the non-embossed films. In the first part of this section, it was assessed whether embossing resulted in a statistically significant improvement in the mechanical properties of the embossed films compared with the non-embossed films.

The reference values were the minimum tensile strength, σ_r_, and relative elongation, ε_r_, obtained for the non-embossed film in the respective material group. The relevant reference value is indicated on each box plot by a red dashed line. The analysis was first conducted with material group and embossing pattern as factors and was subsequently refined by introducing the embossing roller axis distance (RAD) as an additional process factor. In both cases, the Wilcoxon signed-rank test and the sign test were used to assess statistical significance. Results with a *p*-value below 0.05 were considered statistically significant.

In the following subsections, the results are compared across film groups differing in recycled polyethylene content and predominant recycled polyethylene type (LD or HD). The comparison is initially without accounting for additional processing variables and is subsequently extended to include the embossing pattern, film line speed, and embossing roller axis distance. The Brown–Mood median test, followed by pairwise comparisons between the groups, was used to assess differences among the material groups. The Scheirer–Ray–Hare test was used in the factorial analyses to assess the main effects of the factors analysed and their interactions. Where further clarification of the differences among the levels of a given factor was required, the analysis was supplemented with post hoc comparisons using Dunn’s test, with *p*-values adjusted for multiple comparisons. This approach enabled the assessment of both the overall effect of the film material composition and the effects of selected embossing process parameters on the resulting mechanical properties.

### 7.1. Comparison of Embossed and Non-Embossed Films According to Material Group and Embossing Pattern

First, the tensile strength of the embossed films was compared with that of the non-embossed films, with the results grouped according to recycled polyethylene content and embossing pattern. Under this approach, statistically significant increases in tensile strength were identified in only two cases: mean tensile strength, σ_r_, in the machine direction (σ_r_ MD) for films from the 54HDPE group, and mean tensile strength, σ_r_, in the cross direction (σ_r_ CD) for films from the 66HDPE group. For relative elongation, no significant improvement compared with the non-embossed films was found in either direction (MD or CD).

**Figure 4:**
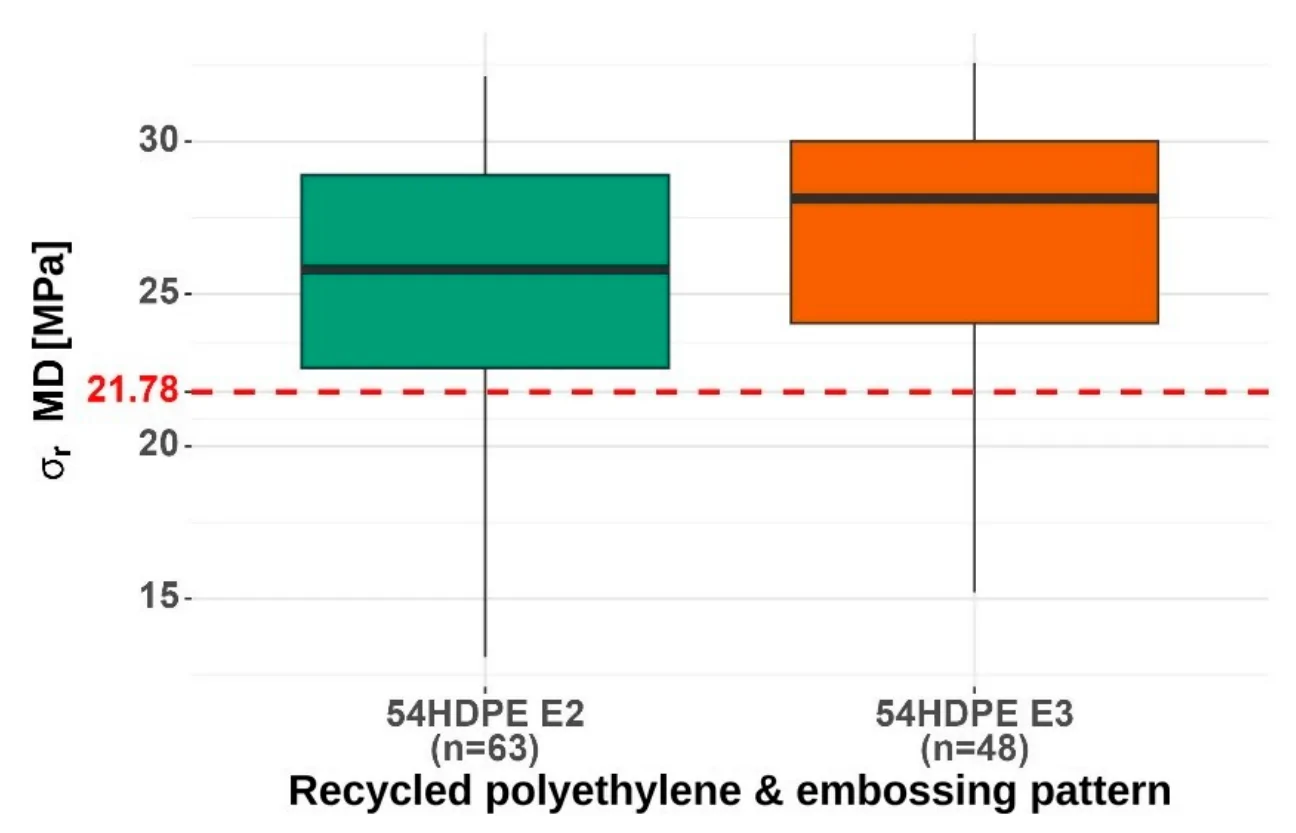
Distribution of mean tensile strength in the machine direction (σ_r_ MD) for embossed films from the 54HDPE group relative to the non-embossed film.

Figure 4 shows the distribution of mean tensile strength in the machine direction, (σ_r_ MD), for films from the 54HDPE group, taking into account embossing patterns E2 and E3. The red dashed line represents the reference value of 21.78 MPa, determined as the minimum tensile strength obtained for the non-embossed film in this material group. The results indicate that, for both embossing patterns analysed, the distribution of tensile strength values was shifted above the reference level, as confirmed by the statistical tests. Table 4 summarizes the statistical test results for the family of hypotheses evaluating significant increases in σ_r_ MD, considering all analyzed recycled polyethylene content levels and embossing patterns relative to the non-embossed control film.

**Figure 5:**
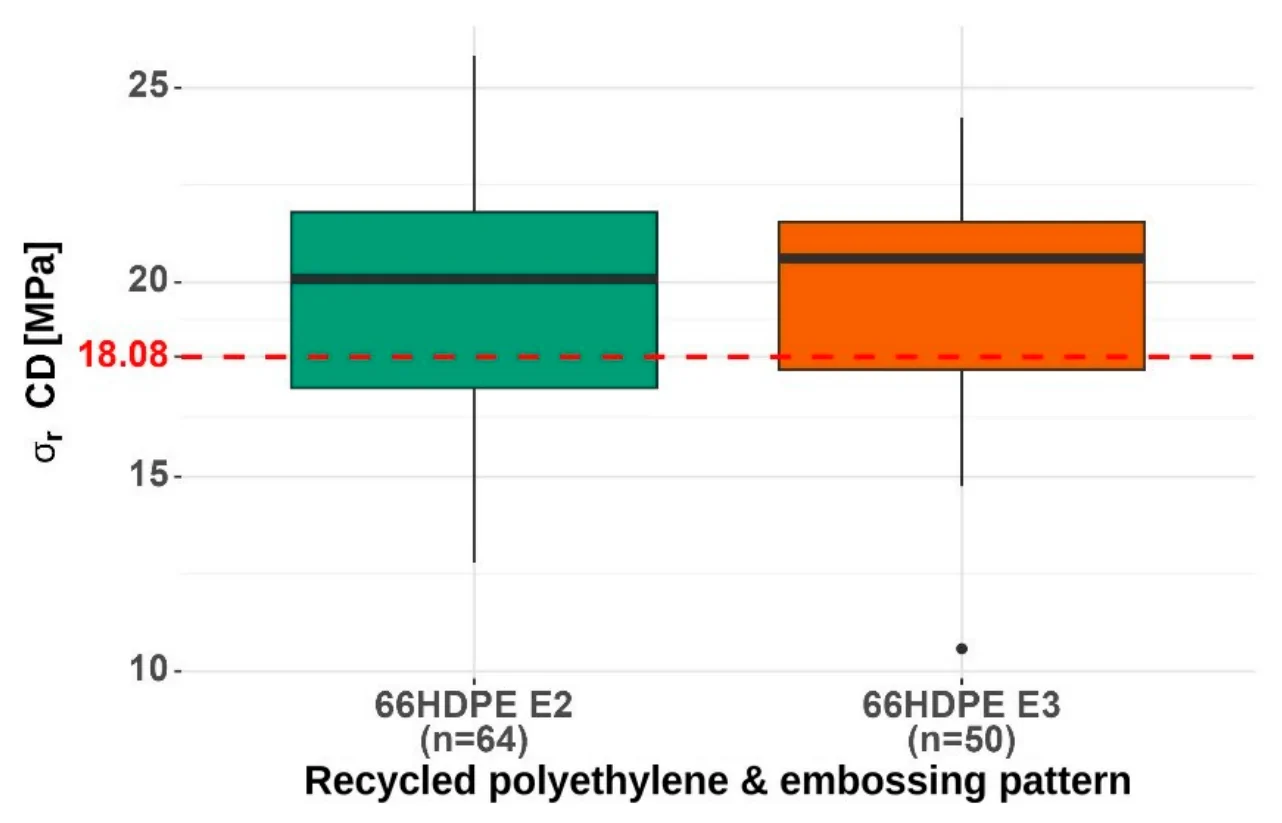
Distribution of the mean tensile strength in the cross direction (σ_r_ CD) for embossed films from the 66HDPE group relative to the non-embossed film.

The results indicate that, for films from the 54HDPE group, the use of the E2 and E3 embossing patterns led to a statistically significant increase in tensile strength, σ_r,_ in the machine direction. In both cases, the results of the Wilcoxon signed-rank test and the sign test led to the same statistical decision, providing consistent evidence of an increase relative to the adopted reference level. It may therefore be concluded that, for films containing approximately 54% recyclate with HDPE as the predominant component, embossing with the E2 and E3 patterns had a beneficial effect on strength in the MD. Figure 5 presents an analogous comparison of tensile strength in the cross direction (σ_r_ CD), for films from the 66HDPE group, taking into account the E2 and E3 embossing patterns. In this case, the reference value, indicated by the red dashed line, was 18.08 MPa. A statistically significant increase in tensile strength, σ_r_ CD, was observed for both embossing patterns analysed.

Table 5 summarizes the statistical test results for the family of hypotheses evaluating significant increases in σ_r_ CD, considering all analyzed recycled polyethylene content levels and embossing patterns relative to the non-embossed control film.

The results presented in Table 5 indicate that, for films from the 66HDPE group, embossing with the E2 and E3 patterns led to a significant increase in tensile strength in the cross direction. This effect was confirmed by both the Wilcoxon signed-rank test and the sign test. This means that, at a higher recycled polyethylene content with a predominance of HDPE in the recyclate fraction, the favourable effect of embossing was evident primarily in the cross direction (CD).

In the remaining cases analysed, no statistically significant increase in tensile strength was found compared with the non-embossed film. This applied in particular to tensile strength in the MD (σ_r_ MD) for the 54LDPE and 66HDPE groups, and to tensile strength in the CD (σ_r_ CD) for the 54LDPE and 54HDPE groups. No statistically significant increase in relative elongation was found in either the machine direction (MD) or the cross direction (CD). These results indicate that the effect of embossing is not universal but depends on the material composition of the film and the direction of the tensile load applied.

### 7.2. Comparison of Embossed and Non-Embossed Films, Taking into Account the Embossing Roller Axis Distance

In the next stage, the analysis was refined by taking into account the embossing roller axis distance (RAD). This made it possible to determine which embossing process settings produced a statistically significant increase in tensile strength compared with the non-embossed film. As in the overall analysis, the reference point was the minimum tensile strength value obtained for the non-embossed film in the given material group. This value is indicated on the box plots by a red dashed line.

In this part of the analysis, the *p*-values obtained from both the Wilcoxon signed-rank test and the sign test are presented. The Wilcoxon signed-rank test was used as the primary tool for assessing the shift in the distribution of results relative to the reference value, whereas the sign test was applied as a supplementary test to verify whether the results predominantly lay above the reference value. A statistically significant increase in tensile strength was assumed only in those cases where the *p*-value was less than 0.05 for both the Wilcoxon signed-rank test and the sign test. This approach reduces the risk of overinterpreting the results, particularly in groups with a small number of observations.

**Figure 6:**
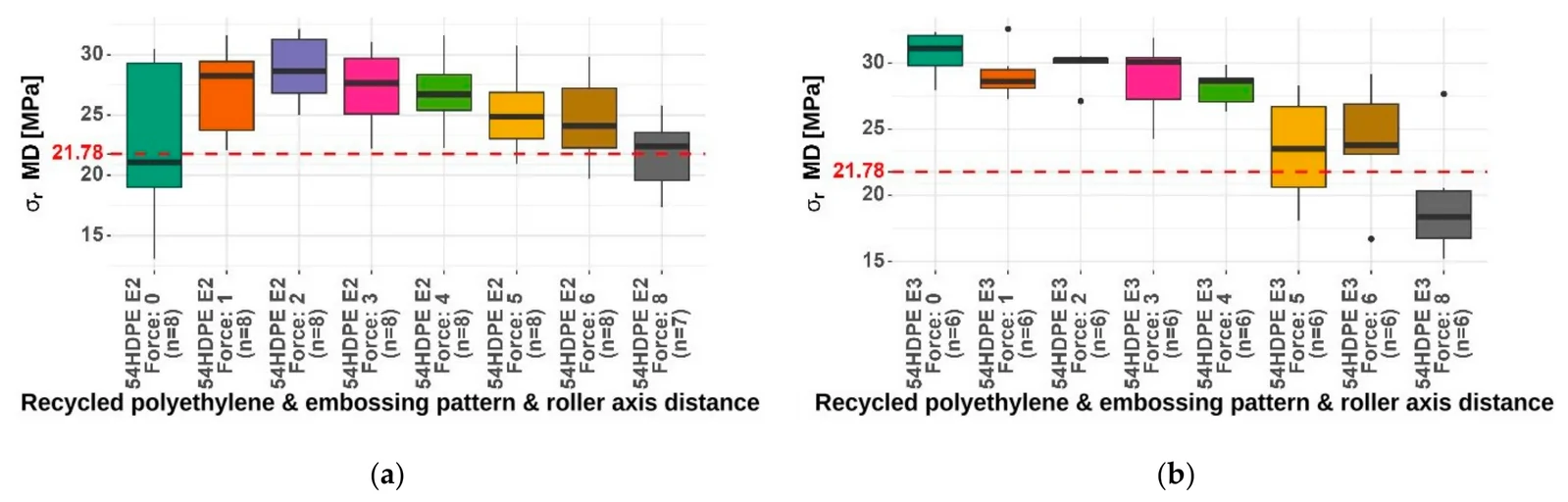
Distribution of mean tensile strength in the machine direction (σ_r_ MD) for films from the 54HDPE group as a function of embossing pattern and embossing roller axis distance (RAD): (**a**) for the E2 embossing pattern; (**b**) for the E3 embossing pattern.

Figure 6 presents the distributions of mean tensile strength in the machine direction for films from the 54HDPE group, grouped by embossing pattern and RAD setting. The reference level was 21.78 MPa. The distributions indicate that, for the E2 and E3 embossing patterns, some combinations of process parameters resulted in a clear shift in tensile strength values above the reference level.

Table 6 summarizes the statistical test results for the family of hypotheses evaluating significant increases in σ_r_ MD, considering all analyzed recycled polyethylene content levels, embossing patterns, and roller axis distance (RAD) settings relative to the non-embossed control film.

Table 6 presents the combinations of factors that yielded the largest effect sizes (includes only those combinations of factors for which the effect size indicated a large effect, defined as an effect size greater than 0.6) in the direction of increased strength relative to the non-embossed film. Despite the high effect sizes observed in the Wilcoxon signed-rank tests and the sign tests, the *p*-values were relatively high and exceeded the conventional significance threshold of 0.05 in most comparisons. Despite the apparently large effect sizes, the results did not provide sufficient statistical evidence to reject the null hypotheses at the 5% significance level. This lack of statistical significance may be partly attributable to the small sample sizes, which limit statistical power, particularly when accounting for multiple comparisons. The results obtained indicate that the favourable effect of embossing on tensile strength in the machine direction (σ_r_ MD) occurred mainly at low and intermediate RAD values, whereas at higher RAD values, corresponding to lower embossing intensity levels, this effect became weaker. However, these findings should be interpreted cautiously and considered exploratory.

**Figure 7:**
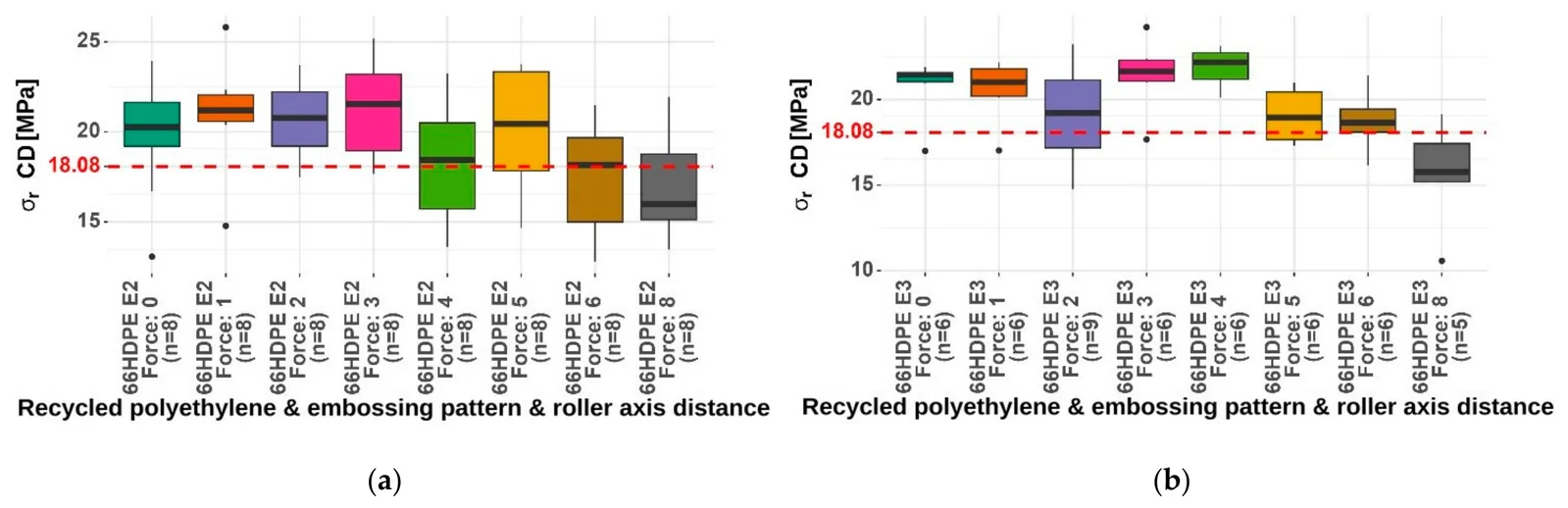
Distribution of mean tensile strength in the cross direction (σ_r_ CD) for films from the 66HDPE group as a function of embossing pattern and embossing roller axis distance (RAD): (**a**) for the E2 embossing pattern; (**b**) for the E3 embossing pattern.

Figure 7 presents the corresponding results for tensile strength in the cross direction (CD) for films from the 66HDPE group. The reference value was 18.08 MPa. The plot indicates that some of the result distributions, particularly for the E2 embossing pattern at lower RAD values and for the E3 embossing pattern at selected settings, were shifted above the reference level. Table 7 presents the *p*-values from the Wilcoxon signed-rank test and the sign test for combinations of embossing pattern and RAD for which the Wilcoxon signed-rank test indicated a significant increase in tensile strength in the cross direction (σ_r_ CD) compared with the non-embossed film.

Table 7 summarizes the statistical test results for the family of hypotheses evaluating significant increases in σ_r_ CD, considering all analyzed recycled polyethylene content levels, embossing patterns, and roller axis distance (RAD) settings relative to the non-embossed control film.

The results presented in Table 7 show that, for films in the 66HDPE group, the effect sizes were consistently high (the table includes only those combinations of factors for which the effect size indicated a large effect, defined as an effect size greater than 0.6) for both the Wilcoxon signed-rank test and the sign test. However, the *p*-values remained high in most comparisons, with Holm-adjusted *p*-values for the Wilcoxon test and sign-test. Consequently, none of the comparisons provided sufficient statistical evidence to confirm a significant increase in tensile strength in the cross direction at the 0.05 significance level. Despite the high effect sizes, the high *p*-values indicate that the observed results do not allow for a statistically conclusive confirmation of improved tensile strength. This discrepancy may be partly attributable to the small sample sizes, which limit statistical power. Therefore, the findings should be interpreted cautiously and considered exploratory, indicating a possible trend towards increased tensile strength rather than a statistically confirmed improvement.

The analysis taking RAD into account confirms that the effect of embossing on tensile strength depends on the embossing intensity. For 54HDPE films, a favourable effect in the machine direction (MD) was observed for the E2 embossing pattern at RAD values from 1 to 5 and for the E3 embossing pattern at RAD values from 0 to 4. For 66HDPE films, a favourable effect in the cross direction (CD) was confirmed for the E2 embossing pattern at RAD values from 1 to 3 and for the E3 embossing pattern at RAD = 4. The lack of confirmed statistical significance across the full range of RAD values indicates that increasing the embossing intensity does not automatically lead to a further improvement in tensile strength. Excessively intensive embossing may reduce the strengthening effect, probably as a result of local weakening of the film structure in the embossed areas.

### 7.3. Comparison of Tensile Strength Among Groups with Different Recycled Polyethylene Contents

The next stage of the analysis involved comparing the results obtained for embossed films belonging to three material groups: 54LDPE, 54HDPE, and 66HDPE. In this part of the analysis, the results were not further subdivided according to embossing pattern, embossing roller axis distance, or film line speed. The aim of the analysis was to determine whether the recycled polyethylene content and its predominant type alone differentiated tensile strength and the percentage values of the parameters analysed. The Brown–Mood median test was used to compare the groups, followed by pairwise comparisons among the 54LDPE, 54HDPE, and 66HDPE groups. The results of the analysis showed that recycled polyethylene content and type significantly differentiated all the parameters analysed: mean tensile strength σ_r_ in the machine direction (MD) and cross direction (CD), as well as relative elongation ε_r_, determined correspondingly in both the machine direction (MD) and transverse direction (CD). The summary results of the global test and the statistically significant pairwise comparisons are presented in Table 8.

**Figure 8:**
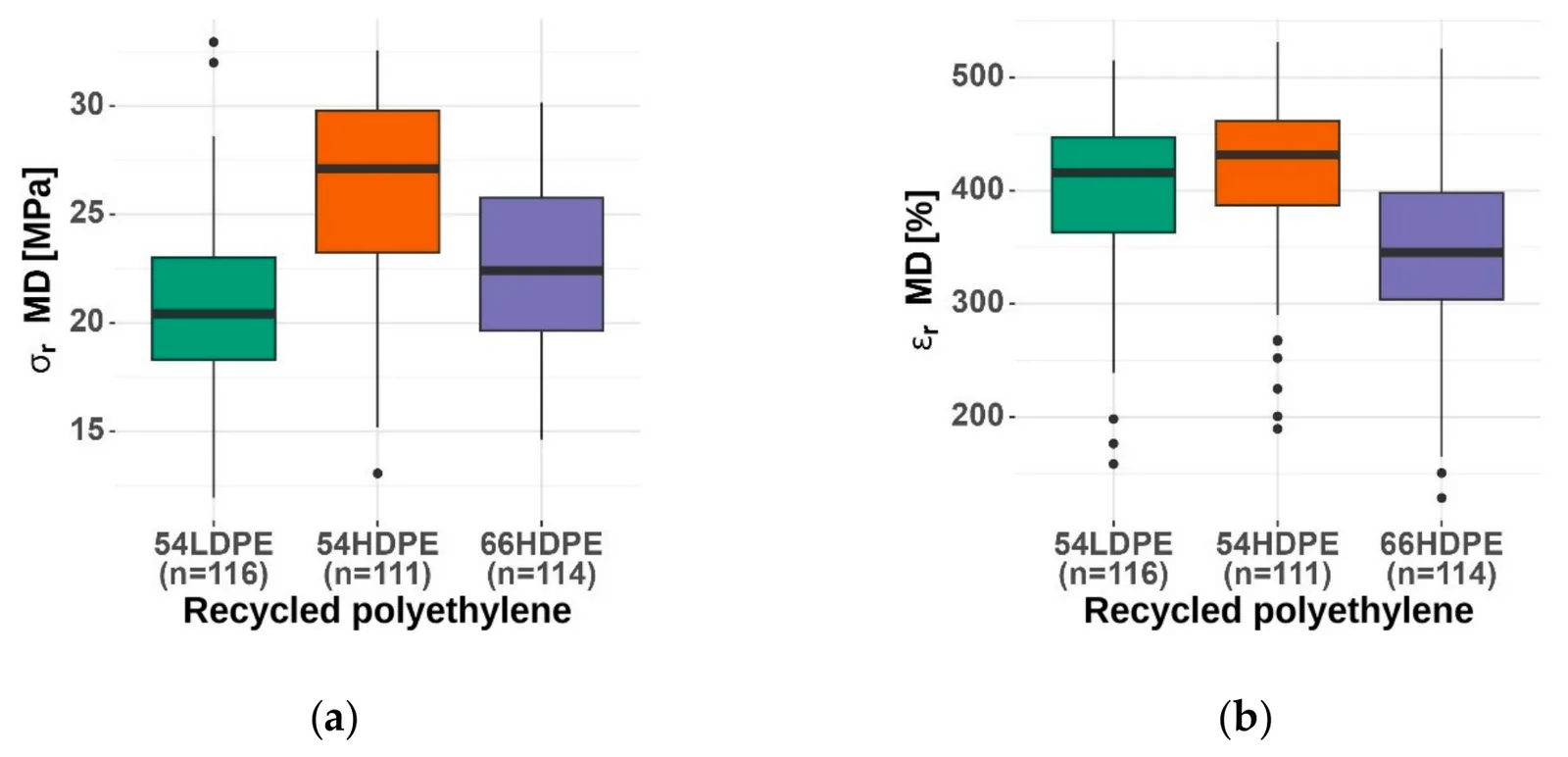
Distributions of mean tensile strength σ_r_ (**a**); and relative elongation ε_r_ (**b**) in the machine direction (MD) for embossed films from the 54LDPE, 54HDPE, and 66HDPE groups.

For tensile strength in the machine direction (σ_r_ MD), the 54HDPE group exhibited the highest values, which were statistically significantly more favourable than those obtained for other groups. This group differed significantly from both the 54LDPE and 66HDPE groups. This indicates that, when measured in the machine direction, the use of films containing approximately 54% recycled polyethylene, with HDPE as the predominant recyclate type, had a favourable effect. At the same time, the results indicate that increasing the recycled polyethylene content to approximately 66% did not lead to any further improvement in tensile strength in the MD. For relative elongation in the machine direction (ε_r_ MD), more favourable results were obtained for the 54LDPE and 54HDPE groups. These two groups did not differ significantly from each other, whereas each differed significantly from the 66HDPE group. This indicates that, for relative elongation in the MD, increasing the recycled polyethylene content to approximately 66% resulted in poorer performance compared with films containing approximately 54% recycled polyethylene. The distributions of σ_r_ MD and ε_r_ MD values for the material groups analysed are presented in Figure 8.

**Figure 9:**
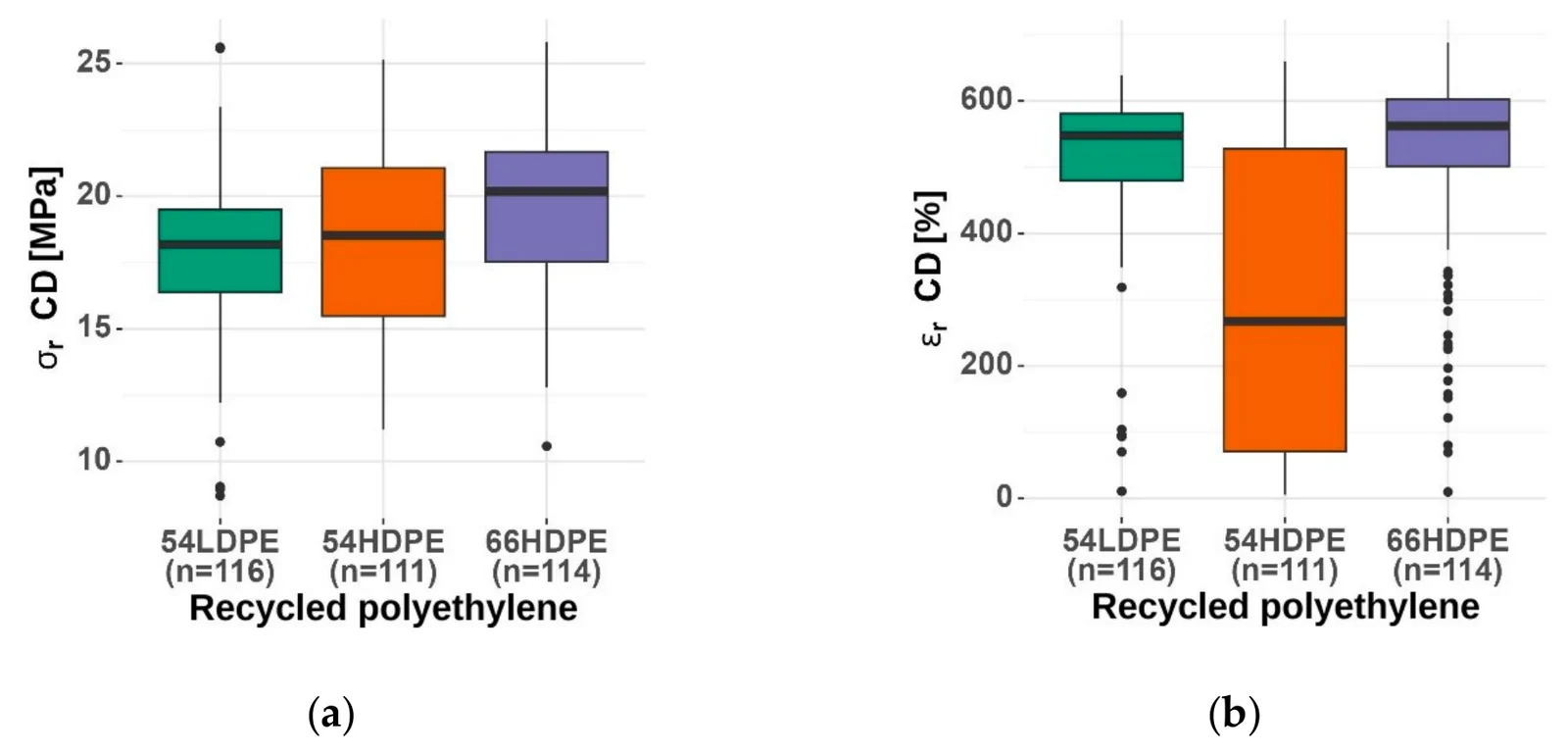
Distributions of mean tensile strength σ_r_ (**a**) and relative elongation ε_r_ (**b**) in the cross direction (CD) for embossed films from the 54LDPE, 54HDPE, and 66HDPE groups.

A different relationship was observed for tensile strength in the cross direction (σr CD). The 66HDPE group exhibited the highest values, which were significantly higher than those obtained for both the 54LDPE and 54HDPE groups. No significant difference was found between the 54LDPE and 54HDPE groups. This indicates that increasing the recycled polyethylene content to approximately 66%, with HDPE as the predominant recyclate type, contributed to an improvement in tensile strength in the cross direction (σ_r_ CD). For relative elongation ε_r_ in the cross direction (ε_r_ CD), more favourable results were obtained for the 54LDPE and 66HDPE groups. These groups did not differ significantly from each other, whereas both yielded significantly better results than the 54HDPE group. This indicates that the effect of material composition on relative elongation in the CD is not determined solely by the HDPE content but also depends on the proportion of recycled polyethylene and, most likely, on the manner in which the film structure responds to deformation in the cross direction. The distributions of mean tensile strength σ_r_, and relative elongation ε_r_ in the cross direction (CD) for the material groups analysed are presented in Figure 9.

In summary, the comparison of the material groups without accounting for additional variables showed that film composition had a significant effect on the mechanical test results. In the machine direction (MD), the 54HDPE group exhibited the highest mean tensile strength σ_r_, whereas in the cross direction (CD), the highest values were obtained for the 66HDPE group. The results expressed as percentages further indicated that the groups with a lower recycled polyethylene content, particularly 54LDPE and 54HDPE, exhibited more favourable relative elongation ε_r_ in the MD, whereas in the CD, favourable results were obtained for the 54LDPE and 66HDPE groups.

### 7.4. Comparison of Mechanical Strength Between Recycled Polyethylene Groups with Respect to Embossing Pattern

Subsequently, an analysis was conducted taking into account two factors: recycled polyethylene content and embossing pattern. The aim of this part of the analysis was to determine whether the differences among the recycled polyethylene content persisted after accounting for embossing pattern geometry and whether an interaction occurred between film composition and embossing pattern.

The Scheirer–Ray–Hare test was used to assess the main effect of recycled polyethylene content and the main effect of embossing pattern. For the interaction between these factors an aligned rank transform analysis of variance was used (Table 9). The results of the Scheirer–Ray–Hare test for the effects of recycled polyethylene content, the effect of embossing pattern and results of the ART ANOVA for the interaction between the two factors are presented in Table 10.

**Figure 10:**
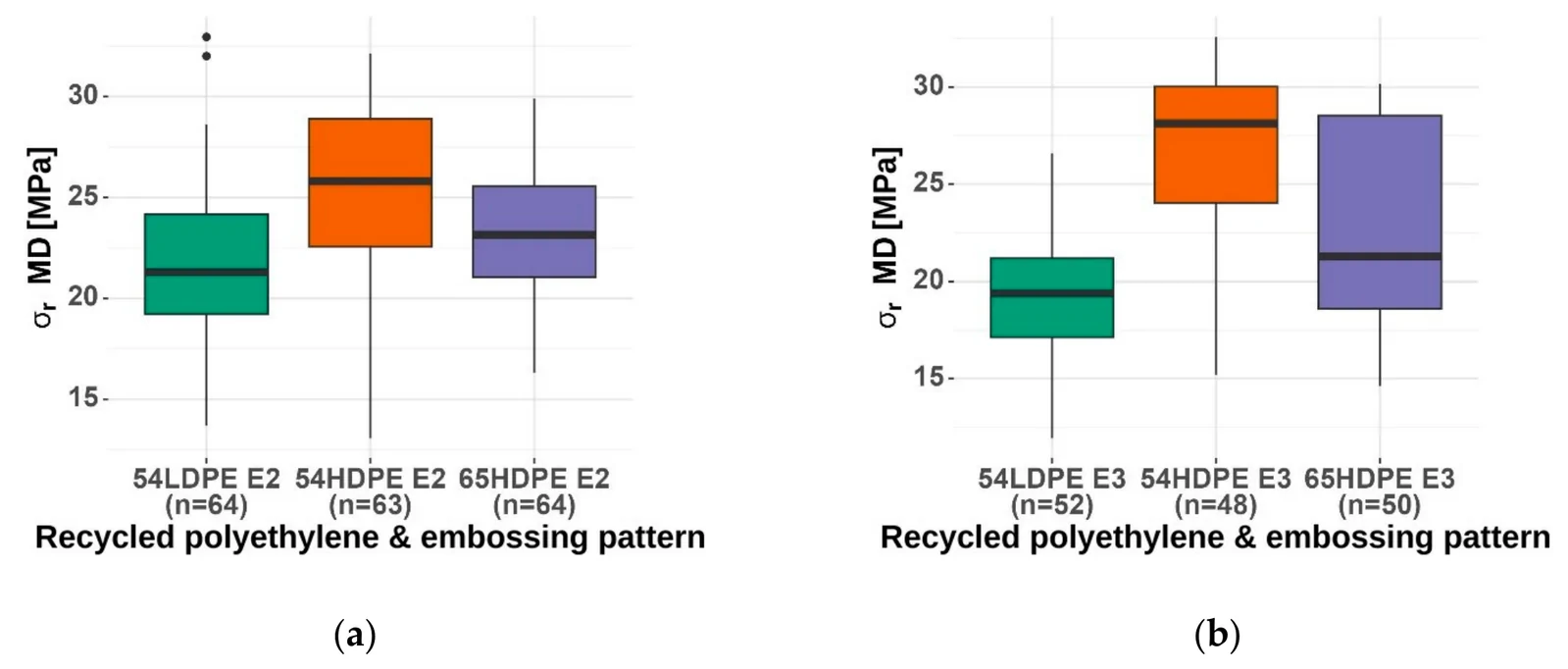
Distribution of mean tensile strength values in the machine direction (σ_r_ MD) for embossed films from the 54LDPE, 54HDPE, and 66HDPE groups, taking into account the E2 (**a**) and E3 (**b**) embossing patterns.

For tensile strength in the machine direction (σ_r_ MD), statistically significant effects of both recycled polyethylene content and embossing pattern were found. The interaction between these factors was also statistically significant. This means that the effect of embossing pattern on tensile strength σ_r_ in the MD depended on the material group of the film. The most favourable results were obtained for the 54HDPE group and, with regard to embossing pattern, for the E2 and E3 variants. The interaction analysis indicated that the 54HDPE E2 and 54HDPE E3 combinations were particularly favourable. This result is consistent with the earlier analysis comparing embossed and non-embossed films, in which a significant increase in tensile strength in the machine direction (MD) was observed specifically for the 54HDPE group with the E2 and E3 embossing patterns. A graphical comparison of the distributions of mean tensile strength σ_r_ in the machine direction (MD) for the material groups and the E2 and E3 embossing patterns is presented in Figure 10.

**Figure 11:**
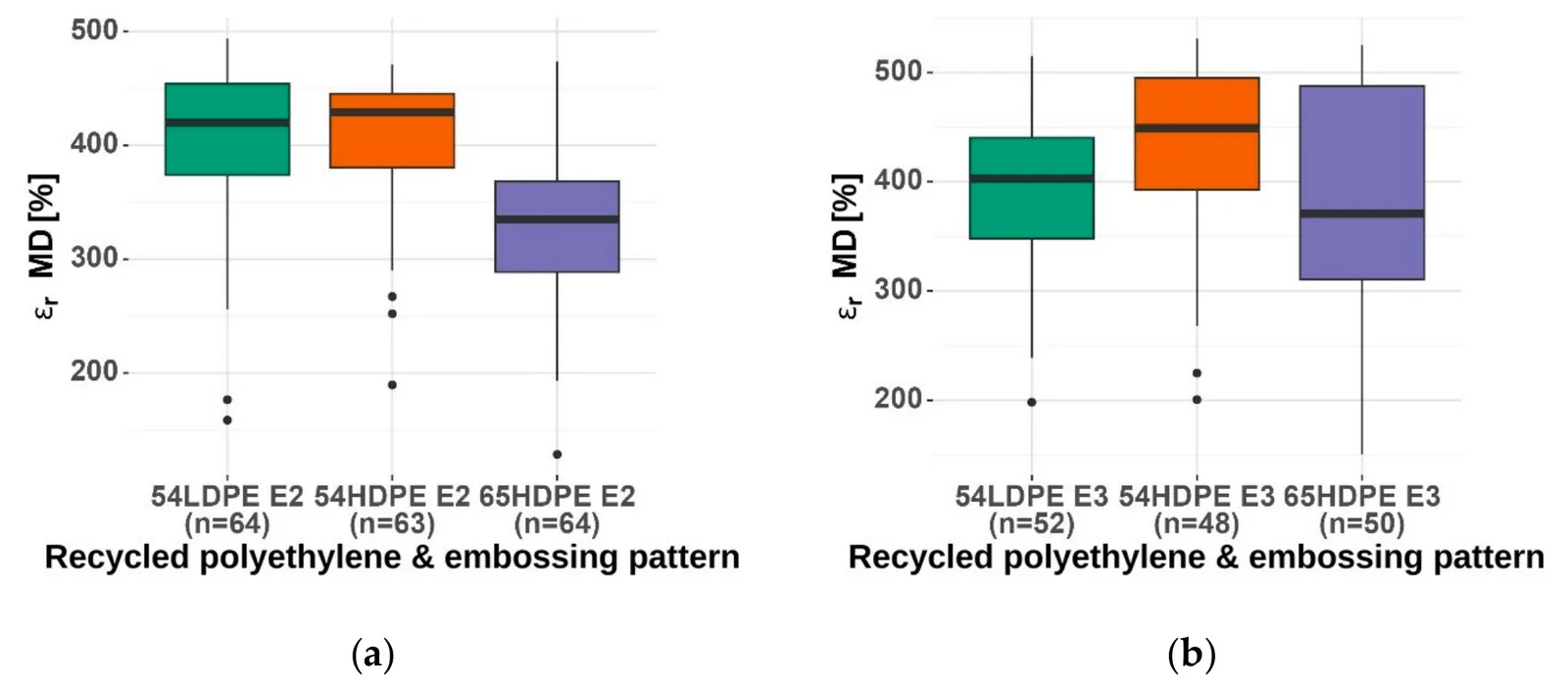
Distribution of relative elongation values in the machine direction (ε_r_ MD) for embossed films from the 54LDPE, 54HDPE, and 66HDPE groups, taking into account the E2 (**a**) and E3 (**b**) embossing patterns.

For relative elongation ε_r_ in the machine direction (MD), statistically significant effects of recycled polyethylene content, embossing pattern, and the interaction between these factors were also found. More favourable results were obtained for the 54LDPE and 54HDPE groups, whereas the 66HDPE group was characterised by lower relative elongation values. Among the variants analysed, favourable results were observed for selected combinations with the E3 embossing pattern, indicating that the improvement in relative elongation ε_r_ in the MD depended not only on the material composition of the film, but also on the embossing pattern. The distributions of relative elongation ε_r_ values in the machine direction for the same combinations of factors are presented in Figure 11.

**Figure 12:**
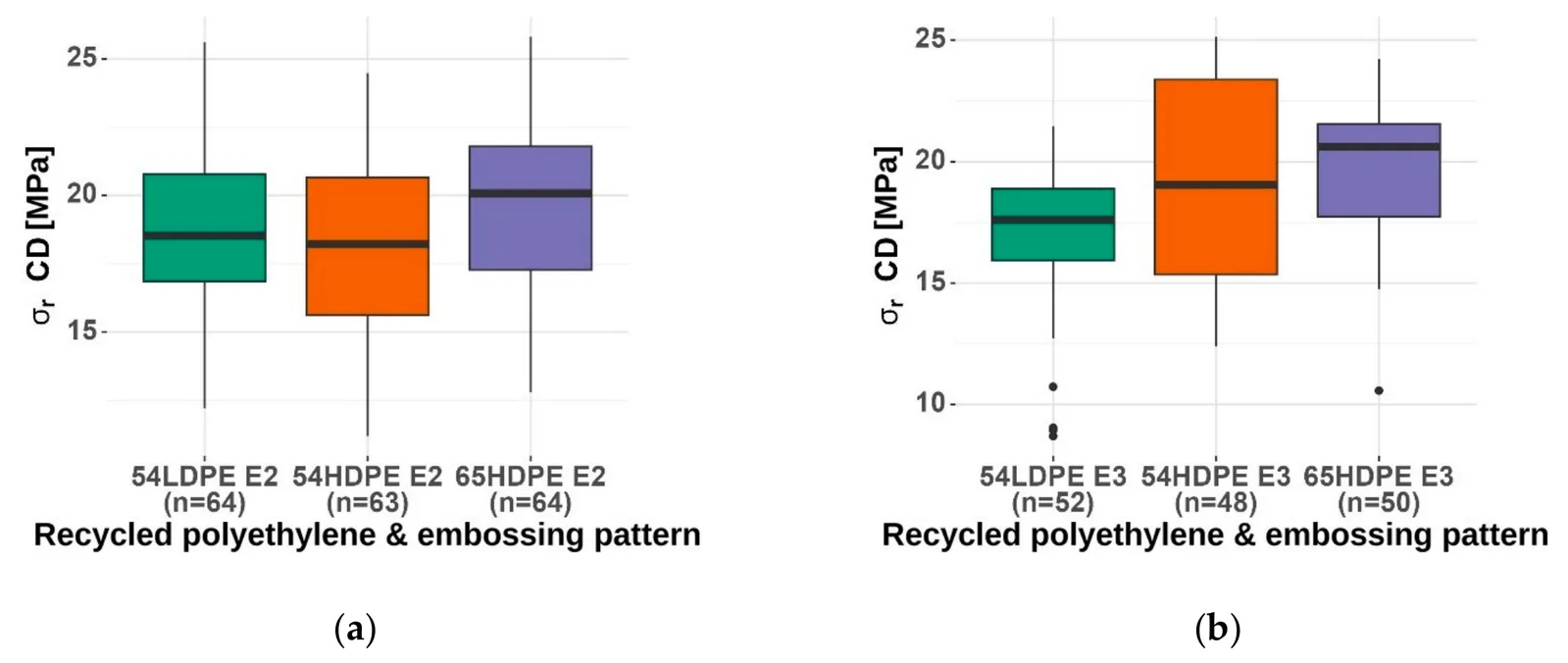
Distribution of mean tensile strength values in the cross (σ_r_ CD) for embossed films from the 54LDPE, 54HDPE, and 66HDPE groups, taking into account the E2 (**a**) and E3 (**b**) embossing patterns.

For mean tensile strength in the cross direction (σ_r_ CD), the effect of recycled polyethylene content was the primary statistically significant factor. The distributions of mean tensile strength values in the cross direction (σ_r_ CD) are presented in Figure 12. The most favourable results were obtained for the 66HDPE group. Neither the effect of embossing pattern nor the interaction between recycled polyethylene content and embossing pattern reached statistical significance. This means that, for absolute tensile strength in the CD, the material composition of the film was the determining factor, whereas the data analysed did not indicate a clear advantage of either of the embossing patterns considered.

**Figure 13:**
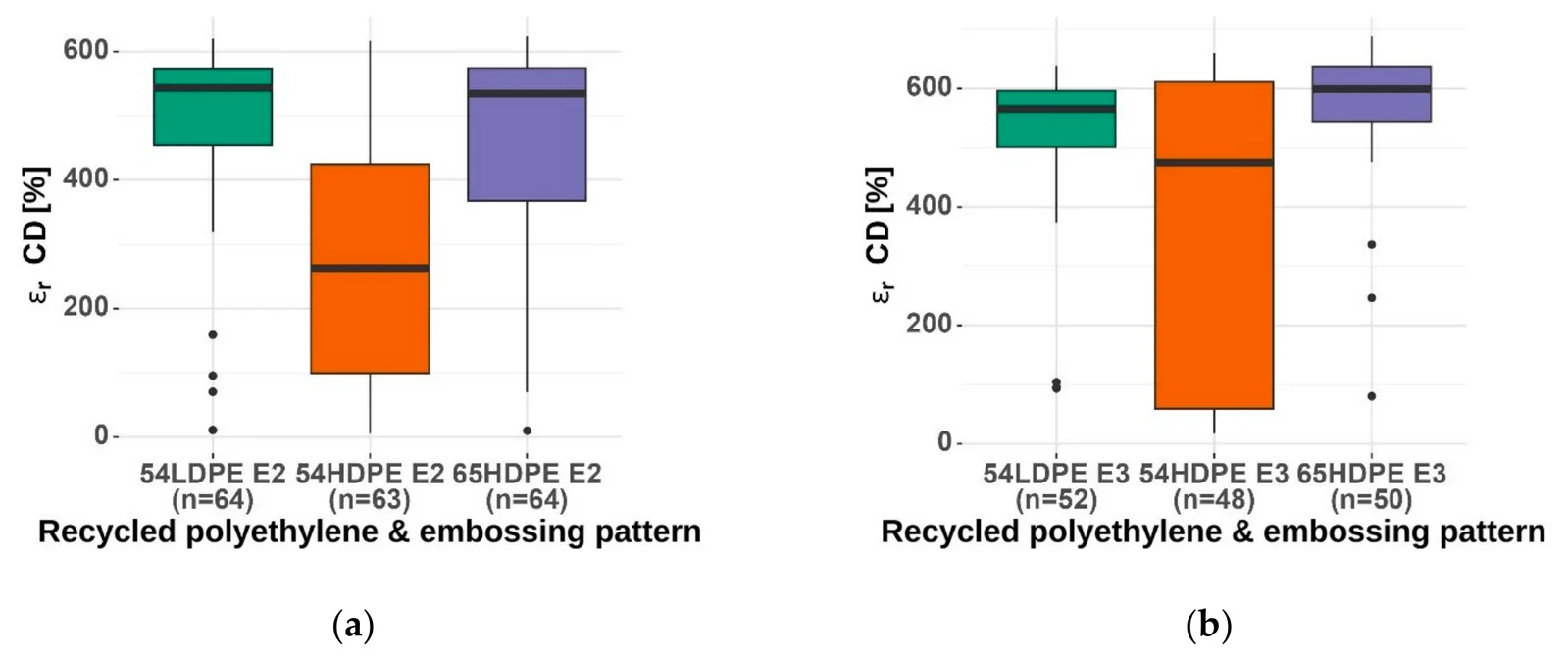
Distribution of relative elongation values in the cross direction (ε_r_ CD) for embossed films from the 54LDPE, 54HDPE, and 66HDPE groups, taking into account the E2 (**a**) and E3 (**b**) embossing patterns.

For relative elongation ε_r_ in the cross direction (CD), statistically significant effects of recycled polyethylene content, embossing pattern, and the interaction between these factors were found. With regard to material group, more favourable results were obtained for the 54LDPE and 66HDPE groups, whereas the 54HDPE group was characterised by lower values. Favourable results were observed for selected combinations of material group and the E3 embossing pattern, confirming that the effect of embossing geometry depended on the material composition of the film. The distributions of relative elongation values in the cross direction (ε_r_ CD) as a function of material group and embossing pattern are presented in Figure 13.

The results obtained indicate that taking the embossing pattern into account provides a better explanation of the variation in film properties, particularly in relation to the loading direction. For mean tensile strength in the machine direction (σ_r_ MD), the effects of material group, embossing geometry, and the interaction between these factors were all statistically significant. The clearest beneficial effect was observed for the 54HDPE group embossed with the E2 and E3 patterns. For mean tensile strength in the cross direction (σ_r_ CD), material group was the decisive factor, with the most favourable results obtained for the 66HDPE group. The analysis of relative elongation values, ε_r_, in the machine and cross directions (ε_r_ MD and ε_r_ CD, respectively) further indicates that the beneficial effects may depend on the specific combination of material composition and embossing pattern.

In summary, the comparative analysis of the material groups confirmed that the material composition of the film is one of the key factors differentiating the test results. Taking the embossing pattern into account further demonstrated that its effect depended on the film type and measurement direction. In the machine direction (MD), the most favourable results were obtained for the 54HDPE group, particularly when combined with the E2 and E3 embossing patterns. For mean tensile strength in the cross direction (CD), the most favourable results were obtained for the 66HDPE group, whereas the effect of the embossing pattern was not statistically significant in the overall analysis.

### 7.5. Comparison of Strength Among Material Groups, Taking into Account the Film Line Speed

In the next stage of the analysis, it was assessed whether the differences in strength among the material groups depended on the film line speed during the embossing process. The analysis was performed for four parameters: mean tensile strength in the machine direction (σ_r_ MD), mean tensile strength in the cross direction (σ_r_ CD), and the corresponding relative elongation values in both directions (ε_r_ MD and ε_r_ CD). Two factors were considered: material group and film line speed (Table 11). In addition, the interaction between these factors was assessed.

**Figure 14:**
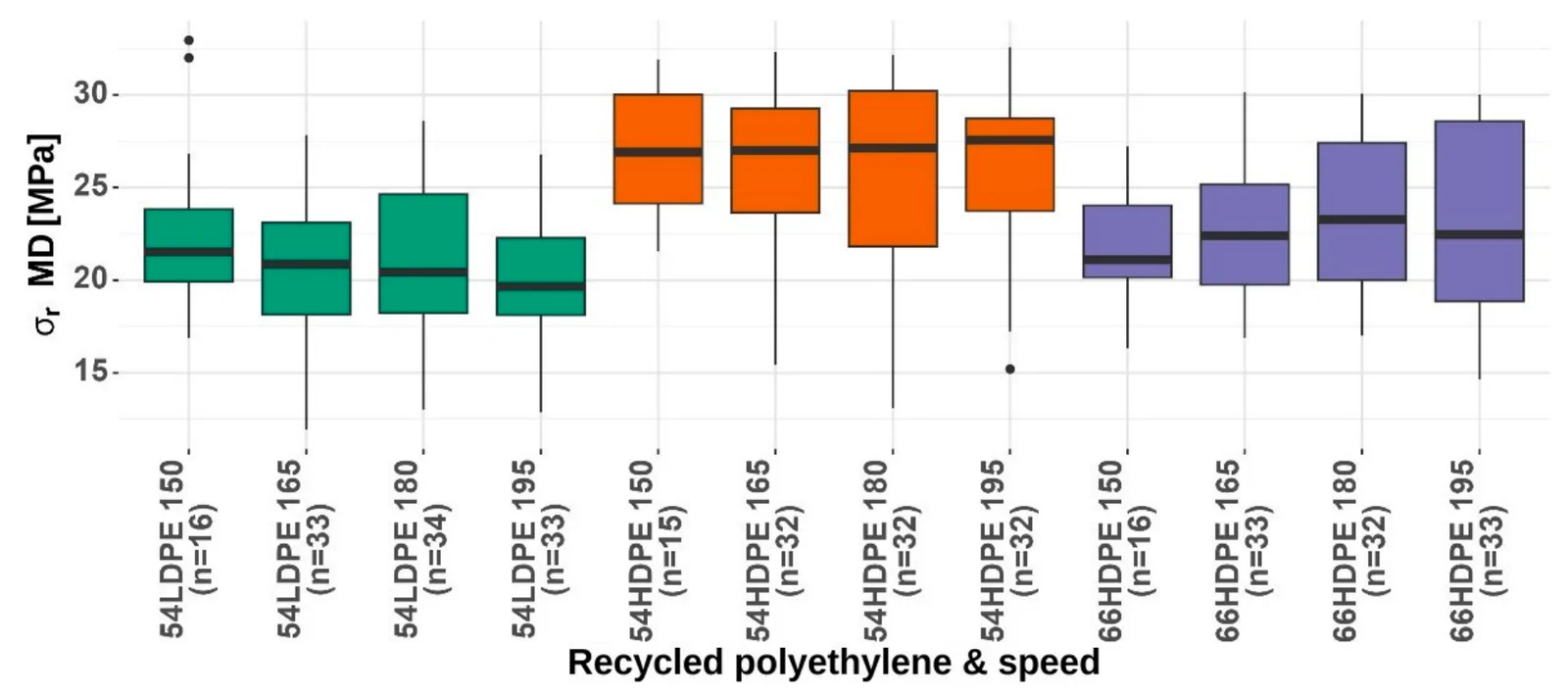
Distribution of mean tensile strength values in the machine direction (σ_r_ MD) for embossed films from the 54LDPE, 54HDPE, and 66HDPE groups, taking into account film line speed.

**Figure 15:**
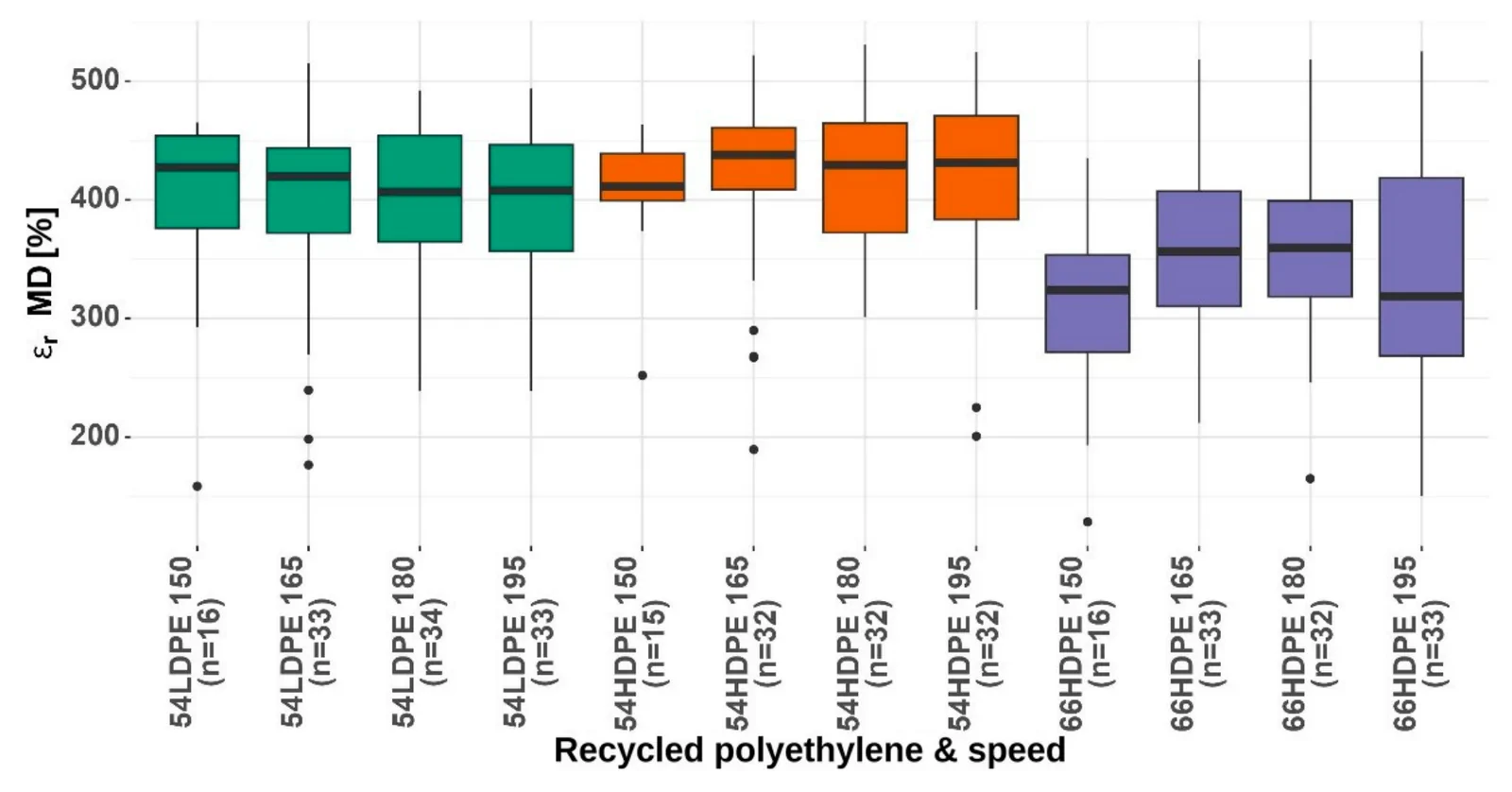
Distribution of relative elongation values in the machine direction (ε_r_ MD) for embossed films from the 54LDPE, 54HDPE, and 66HDPE groups, taking into account film line speed.

**Figure 16:**
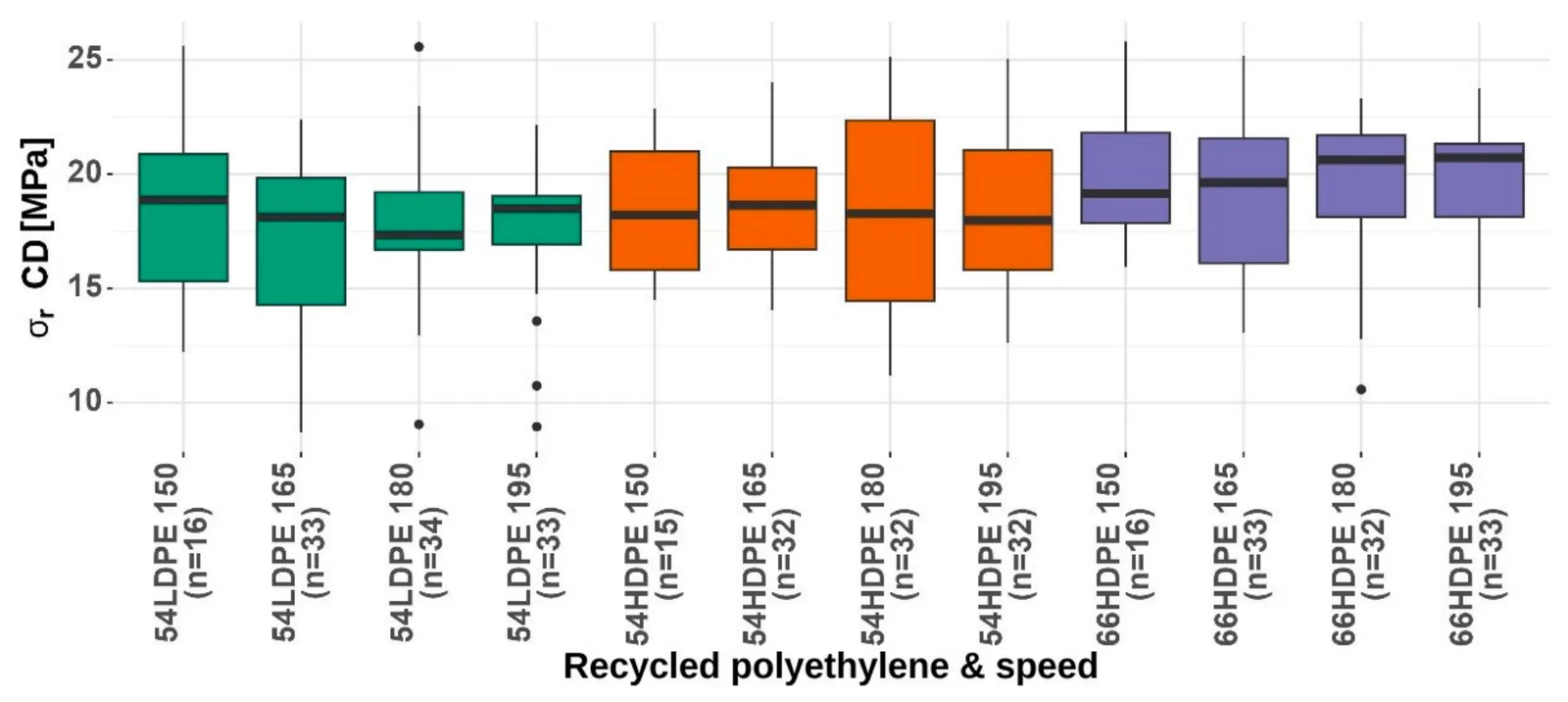
Distribution of mean tensile strength values in the cross direction (σ_r_ CD) for embossed films from the 54LDPE, 54HDPE, and 66HDPE groups, taking into account the film line speed.

**Figure 17:**
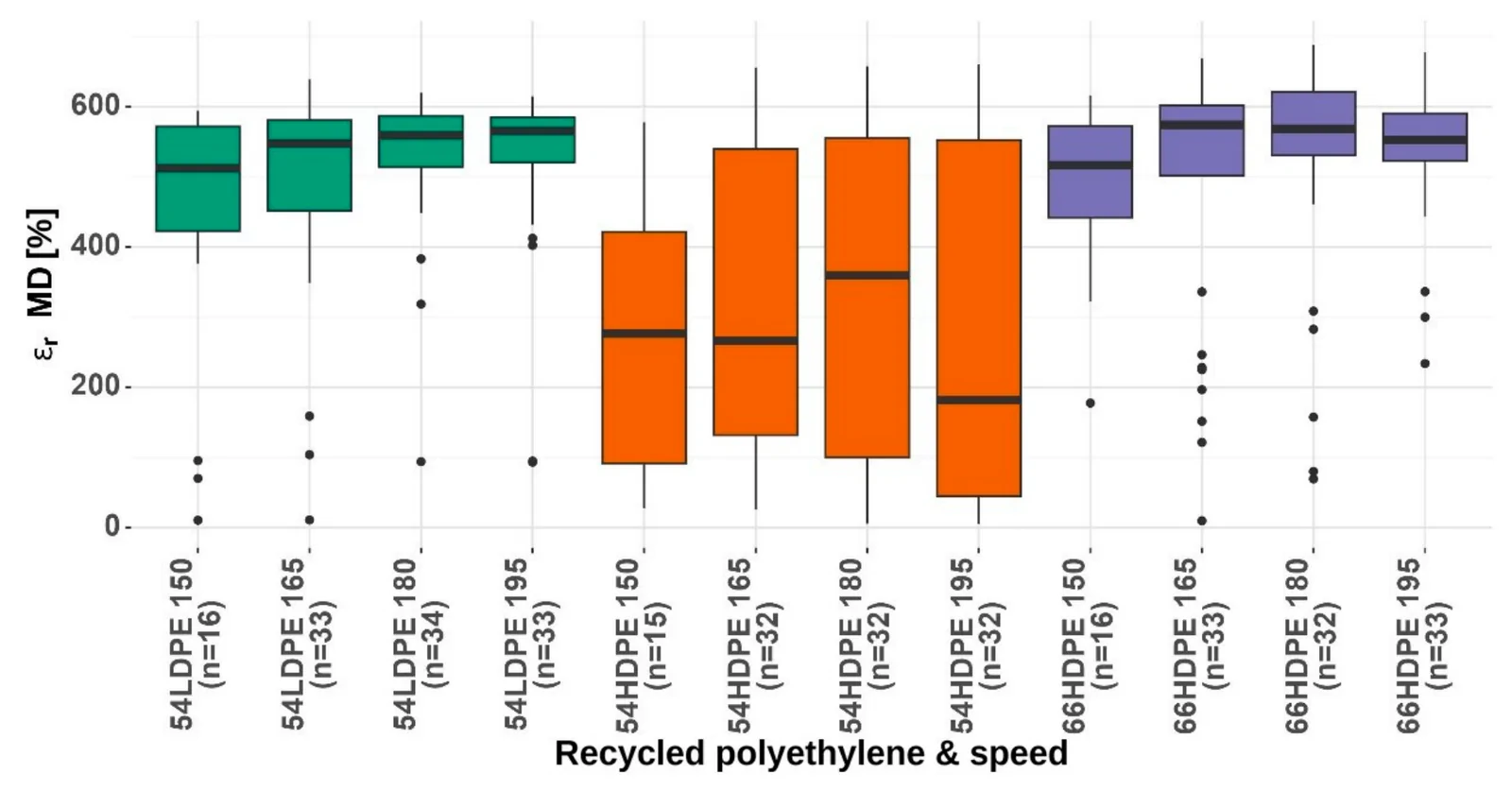
Distribution of relative elongation values in the cross direction (ε_r_ CD) for embossed films from the 54LDPE, 54HDPE, and 66HDPE groups, taking into account the film line speed.

The results of the Scheirer–Ray–Hare test for the effects of material group and film line speed, and results of the ART ANOVA for the interaction between these two factors are presented in Table 12. The distributions of the parameters analysed as a function of material group and film line speed are presented in Figure 14, Figure 15, Figure 16 and Figure 17.

The results of the Scheirer–Ray–Hare test showed that, for all the parameters analysed, the effect of material group was statistically significant. However, neither a significant effect of film line speed nor a significant interaction between material group and film line speed was found. This means that differences among the material groups were evident regardless of the process speed used, whereas changing the speed within the range analysed did not produce statistically significant differences in the results.

For mean tensile strength in the machine direction (σ_r_ MD), the 54HDPE group exhibited the highest values, which were significantly higher than those obtained for both the 54LDPE group and the 66HDPE group. The values obtained for the 66HDPE group were also significantly higher than those obtained for the 54LDPE group. This means that, in terms of absolute tensile strength in the MD, the most favourable results were obtained for films containing approximately 54% recycled polyethylene, with HDPE as the predominant recyclate type. For relative elongation in the machine direction (ε_r_ MD), more favourable values were obtained for the 54LDPE and 54HDPE groups. These groups did not differ significantly from each other, whereas both exhibited significantly higher values than the 66HDPE group. This indicates that increasing the recycled polyethylene content to approximately 66% did not improve relative elongation in the MD but instead resulted in lower values than those obtained for the groups containing approximately 54% recycled polyethylene.

For tensile strength in the cross direction (σ_r_ CD), the most favourable results were obtained for the 66HDPE group. This group differed significantly from the 54LDPE and 54HDPE groups, whereas no significant differences were found between the 54LDPE and 54HDPE groups. This means that, in the CD, the higher recycled polyethylene content with a predominance of HDPE in the recyclate fraction was the determining factor. For relative elongation in the cross direction (ε_r_ CD), more favourable values were obtained for the 54LDPE and 66HDPE groups. These groups did not differ significantly from each other, whereas both exhibited significantly higher values than the 54HDPE group. This result indicates that the relative elongation in the CD depended not only on the HDPE content, but also on the relationship between film composition and the reference level adopted for the given material group.

The results presented in Table 12 and Figure 14, Figure 15, Figure 16 and Figure 17 indicate that, within the range analysed, film line speed did not have a statistically significant effect on the results. It may therefore be concluded that, in this analysis, differences in mechanical properties were determined primarily by the material composition of the film rather than by film line speed. From a technological perspective, this means that the choice of material group had a greater effect on mechanical properties than changes in film line speed within the range investigated.

### 7.6. Comparison of Mechanical Properties (Strength) Among Material Groups, Taking into Account the Embossing Roller Axis Distance

Next, an analysis was performed in which the embossing roller axis distance (RAD), was considered alongside the recycled polyethylene content. Adjusting the distance between the embossing rollers affects the relative position of the elements that form the embossing pattern, and thus the shape and dimensions of the embossing created on the film being processed. Factor levels with very few observations were excluded from the analysis to reduce the risk of misinterpreting the results.

In contrast to the analysis involving film line speed, both the effect of recycled polyethylene content and the effect of RAD were statistically significant. However, no significant interaction between recycled polyethylene content and RAD was found. This means that both the material composition of the film and the embossing intensity affected the mechanical test results (Table 13), but the effect of RAD was similar across the material groups analysed.

The results of the Scheirer–Ray–Hare test for the effects of material group and embossing roller axis distance, and results of the ART ANOVA for the interaction between these two factors are presented in Table 14.

**Figure 18:**
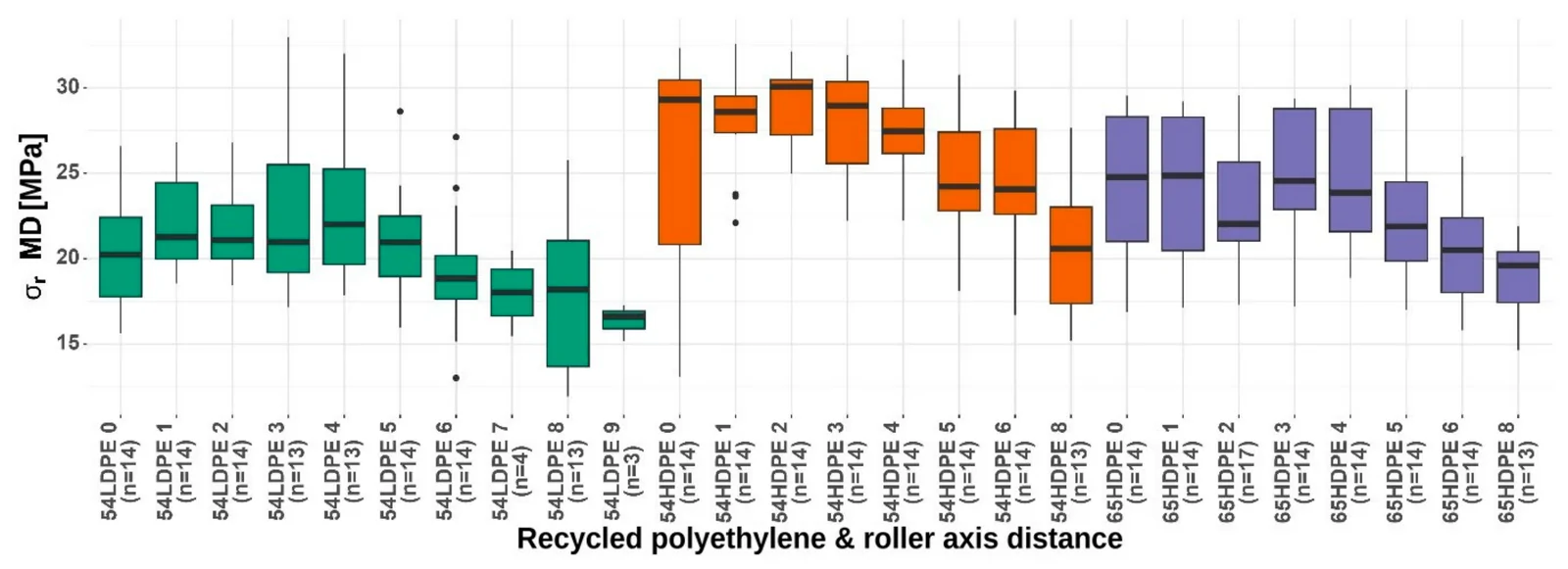
Distribution of mean tensile strength values in the machine direction (σ_r_ MD) for embossed films from the 54LDPE, 54HDPE, and 66HDPE groups, taking into account the embossing roller axis distance (RAD).

**Figure 19:**
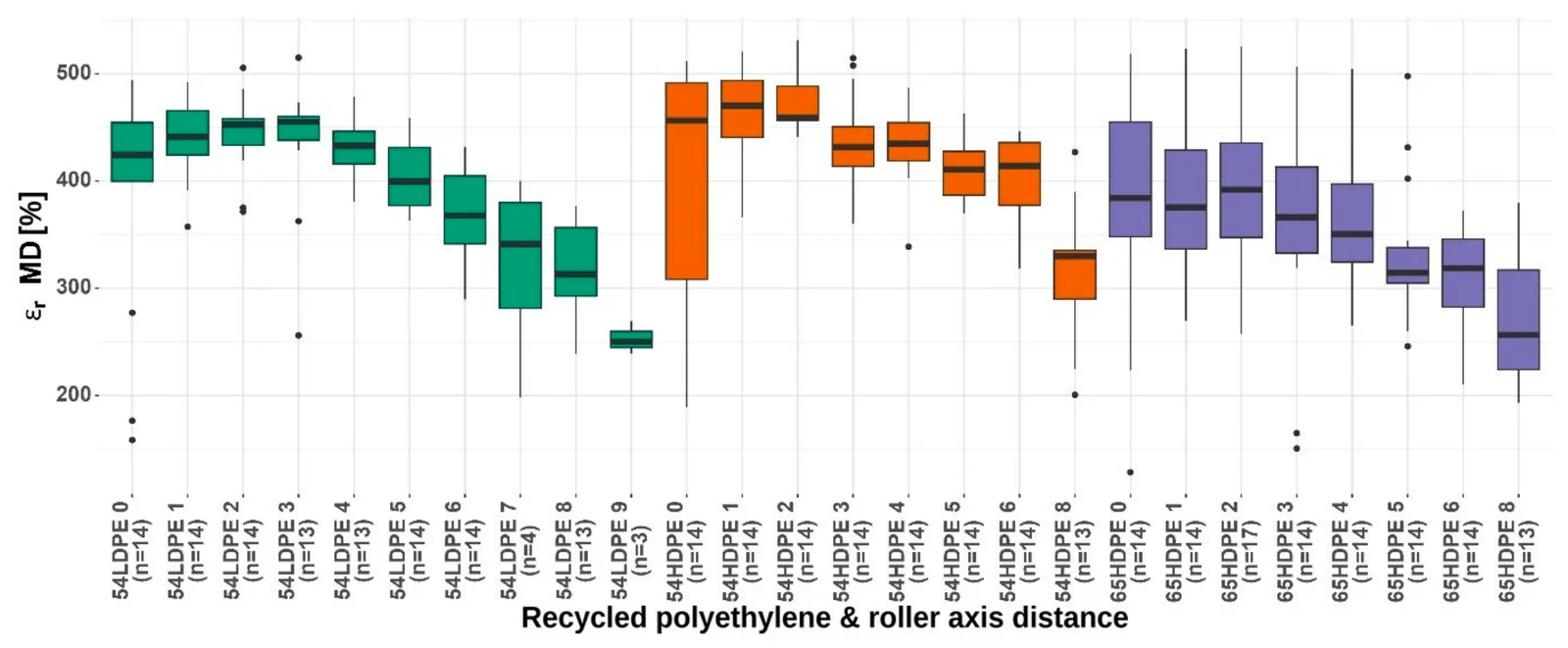
Distribution of relative elongation values in the machine direction (ε_r_ MD) for embossed films from the 54LDPE, 54HDPE, and 66HDPE groups, taking into account the embossing roller axis distance (RAD).

**Figure 20:**
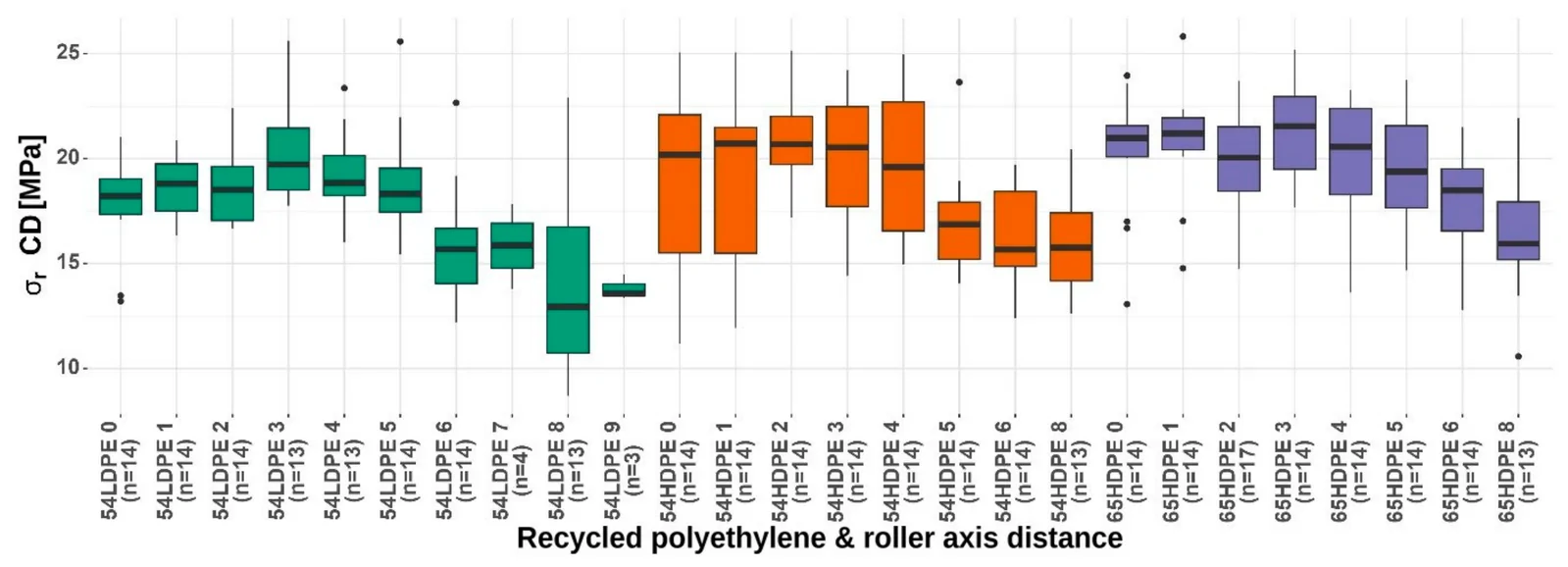
Distribution of mean tensile strength values in the transverse direction (σ_r_ CD) for embossed films from the 54LDPE, 54HDPE, and 66HDPE groups, taking into account the embossing roller axis distance.

**Figure 21:**
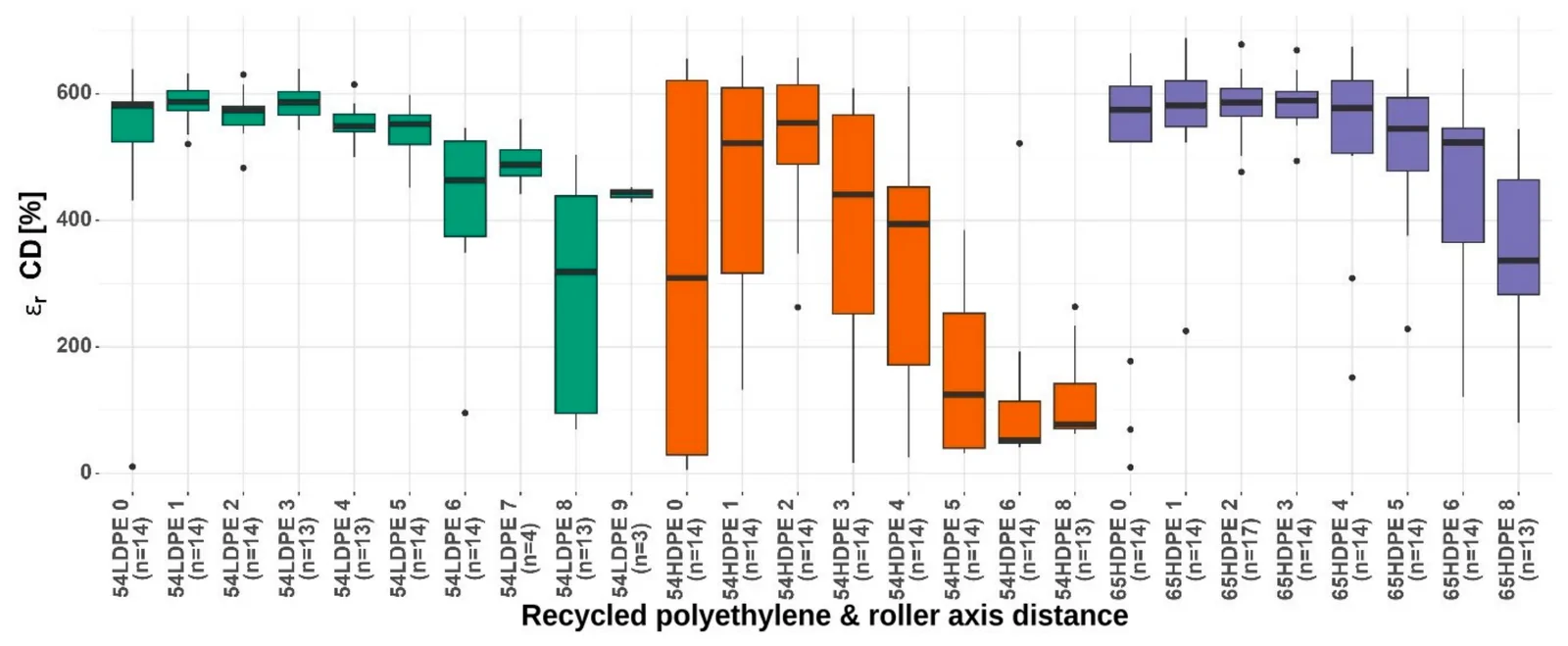
Distribution of relative elongation values in the cross direction (ε_r_ CD) for embossed films from the 54LDPE, 54HDPE, and 66HDPE groups, taking into account the embossing roller axis distance (RAD).

The distributions of the parameters analysed as a function of material group and RAD are presented in Figure 18, Figure 19, Figure 20 and Figure 21. For tensile strength in the machine direction (σ_r_ MD), the most favourable results were again obtained for the 54HDPE group. The significant effect of RAD indicates that embossing intensity influenced tensile strength. The most favourable results were obtained at RAD settings ranging from 0 to 5. At higher RAD settings, particularly at RAD = 8, the results were poorer. This means that increasing the roller axis distance, and thereby reducing embossing intensity, did not lead to any further improvement in tensile strength in the MD but could weaken the reinforcing effect.

For relative elongation in the machine direction (ε_r_ MD), more favourable results were obtained for the 54LDPE and 54HDPE groups. The significant effect of RAD indicates that more favourable relative elongation values were obtained mainly at RAD levels ranging from 0 to 4. Higher RAD settings were less favourable, confirming the observation that excessively intense embossing may reduce the effectiveness of the embossing process.

For tensile strength in the cross direction (σ_r_ CD), the most favourable results were obtained for the 66HDPE group. As in the tensile strength measurements in the machine direction, the effect of RAD was also significant. Favourable results were obtained at RAD settings from 0 to 4, whereas higher RAD settings were associated with poorer results. This indicates that, in the cross direction (CD), the beneficial effect of embossing requires a moderate embossing intensity.

For relative elongation in the cross direction (ε_r_ CD), more favourable results were obtained for the 54LDPE and 66HDPE groups. In this case, the favourable RAD range was narrower and included settings of 1, 2, and 3. This means that the relative improvement in properties in the cross direction (CD) was particularly sensitive to the embossing roller axis distance setting. An embossing intensity that was either too low or too high did not produce results as favourable as those obtained with intermediate settings.

The absence of a significant material group × RAD interaction indicates that the effect of the embossing roller axis distance did not differ significantly among the material groups analysed. In other words, although the individual material groups differed in their overall strength levels, the general direction of the RAD effect was similar: low and intermediate RAD settings were generally favourable, whereas higher settings were associated with poorer results. This is an important technological finding, as it indicates that optimisation of the embossing process should not involve maximising the embossing intensity. The best results were obtained at moderate embossing roller axis distance settings.

In summary, the analysis taking into account film line speed and RAD showed that film line speed did not significantly differentiate the strength results, whereas the embossing roller axis distance was a statistically significant factor for all the parameters analysed. However, the material composition of the film remained the primary factor: the 54HDPE group produced the most favourable results in the machine direction (MD), whereas the 66HDPE group was the most favourable in the cross direction (CD). The results presented in Table 14 and Figure 18, Figure 19, Figure 20 and Figure 21 confirm that low and intermediate RAD settings were generally the most favourable, whereas increasing the RAD, and thereby reducing embossing intensity did not lead to any further improvement in the mechanical properties.

Additionally, after completing the analyses presented in Section 7.1, Section 7.2, Section 7.3, Section 7.4, Section 7.5 and Section 7.6, Aligned Rank Transform Analysis of Variance was performed to explore potential multi-factor interactions among material group, embossing pattern, and embossing roller axis distance (RAD). For the response variables evaluated (σ_r_ MD, σ_r_ CD, ε_r_ MD, and ε_r_ CD), none of the three-way interaction terms reached statistical significance at *α* = 0.05. Given the exploratory nature of these higher-order interaction tests, these non-significant outcomes indicate that strong three-way interactions were not detected within the limits of the present sample size and variability, though subtle joint factor effects cannot be entirely ruled out. The interpretation therefore focused on the main effects and the significant relationships identified in the tests presented above.

## 8. Summary and Interpretation of the Statistical Analysis Results

This section summarises the key results of the statistical analysis presented in Section 7. The summary includes both a comparison of embossed films with non-embossed films and a comparison of the results obtained for the embossed films, taking into account material group, embossing pattern, film line speed, and embossing roller axis distance (RAD). A significance level of α = 0.05 was adopted in all analyses.

Table 15 presents selected combinations of material group (recycled polyethylene content), embossing pattern, and embossing roller axis distance (RAD) for which large effect sizes (effect size > 0.6) were observed in the direction of improved mechanical properties relative to the corresponding non-embossed films. Given the high *p*-values obtained in the statistical tests, these combinations are presented as exploratory findings rather than statistically confirmed improvements. The purpose of this analysis is to identify potentially relevant embossing conditions for further investigation, not to establish definitive evidence of increased film strength.

Based on the data presented in Table 15, it can be concluded that embossing did not result in a universal increase in film strength irrespective of the material composition and process parameters. Improvement in the mechanical properties tested, relative to the non-embossed film, was confirmed only for selected combinations. The analysis of material group and embossing pattern identified statistically significant improvements in tensile strength in the machine direction for the 54HDPE group with embossing patterns E2 and E3, and in the cross direction for the 66HDPE group with embossing patterns E2 and E3. However, when the analysis was extended to specific RAD levels, these improvements did not remain statistically significant after correction of *p*-values using the Holm procedure. Nevertheless, high effect size values (greater than 0.6) were observed for selected combinations, suggesting potentially meaningful strengthening effects that may not have reached statistical significance due to the small sample sizes and the resulting limited statistical power. These findings should therefore be interpreted as exploratory and do not provide sufficient evidence to confirm statistically significant strengthening at individual RAD levels. For relative elongations in the machine and cross directions (ε_r_ MD and ε_r_ CD, respectively), no statistically significant improvement relative to the non-embossed film was confirmed.

Table 16 presents a summary of the comparative analyses conducted exclusively for the embossed films. The table includes the factors that were found to have a significant effect on the variation in the results, together with the levels of these factors associated with more favourable values of the parameters analysed.

The results presented in Table 16 confirm that the material composition was the primary factor differentiating the mechanical properties of the films. For tensile strength in the machine direction (σ_r_ MD), the most favourable results were obtained for films from the 54HDPE group, whereas in the cross direction, the most favourable results were obtained for films from the 66HDPE group. This indicates that the effect of the recycled polyethylene content and predominant recyclate type depended on the tensile loading direction applied. The effect of embossing pattern was statistically significant primarily for tensile strength in the machine direction and relative elongation in both directions. For tensile strength in the machine direction (σ_r_ MD), favourable results were associated with the E2 and E3 embossing patterns, particularly in the 54HDPE group. For tensile strength in the cross direction (σ_r_ CD), the effect of embossing pattern was not statistically significant in the overall analysis, indicating that the material composition of the film was the decisive factor. For relative elongation, favourable results were obtained for selected combinations involving the E3 embossing pattern; however, these results should be interpreted in conjunction with the material composition of the film.

Within the range analysed, the film line speed did not have a statistically significant effect on the mechanical properties tested. This indicates that changes in the embossing process speed were not a decisive factor in determining the resulting mechanical performance. By contrast, the embossing roller axis distance (RAD) had a statistically significant effect. Favourable results were obtained mainly at low and intermediate RAD settings, whereas higher settings, corresponding to lower embossing intensity, were associated with poorer results. These findings may indicate that excessive embossing causes local weakening of the film structure.

The interaction analysis showed that the relationship between the film material composition and embossing pattern was significant, particularly for tensile strength in the machine direction and relative elongation in the cross direction. In the exploratory multi-factor analysis, no higher-order interactions involving all factors simultaneously reached statistical significance, suggesting that two-way main effects and pairwise combinations accounted for the primary variation observed in the dataset. Overall, it can be concluded that the most favourable results for the measured static tensile properties were achieved for films containing recycled polyethylene with a predominance of HDPE, embossed with the E2 or E3 patterns at moderate embossing intensity.

## 9. Final Remarks and Conclusions

Experimental tests of the static tensile strength and relative elongation of embossed three-layer films containing different proportions of recycled polyethylene were conducted under industrial conditions over a three-year period. The study demonstrated the importance of selecting an appropriate material formula and process parameters in determining the mechanical strength of the film. The conclusions may be considered in the following contexts:

- General technological context

The embossing process investigated should be regarded as an industrial film-surface structuring process in which two rollers transfer a geometric pattern onto a three-layer film containing different proportions of recycled PE. The underlying mechanism is not limited to producing a visible surface pattern. Within the roller contact zone, local stress fields and thickness variations develop, accompanied by possible macromolecular reorientation and local densification or stretching of the material; in extreme cases, local loss of material integrity may also occur.

A distinctive feature of the present study is its greater emphasis on aspects that are often overlooked in conventional laboratory investigations of mechanical properties: the use of a three-layer film, the achievement of the required high recycled polyethylene content, and processing conditions closely resembling those encountered in actual production. This approach is important because, in industrial practice, critical considerations include not only maximum strength values but also process stability, the reproducibility of the required functional properties throughout the manufactured product—in this case, along both the length and width of the extruded film tube—the ability to maintain film line speed, and compliance with the technological requirements for both the machine direction (MD) and the cross direction (CD).

The results obtained indicate that embossing cannot be regarded as a universal method for reinforcing all types of film. Rather, it is a processing operation that can improve static tensile strength for selected combinations; no statistically significant improvement in relative elongation relative to the non-embossed reference films was confirmed. Such tensile-strength increases occur only when the material composition, embossing pattern geometry, and embossing intensity form a favourable combination. This interpretation is consistent with findings reported in the literature on patterned PE films, in which pattern depth, processing speed, temperature, and mechanical properties have been shown to be interrelated [1].

- Material context

The material group was the factor that most strongly differentiated the results for the six investigated formulas at nominal recycled-polyethylene contents of 54% and 66%. This indicates that the measured static tensile strength and relative elongation depends on the combined characteristics of the formula as well as the embossing conditions. The comparisons do not isolate the influence of recycled content from recycled-polyethylene type or other differences between formulas. The most favourable tensile-strength results in the machine direction (MD) were obtained for the 54HDPE group, whereas those in the cross direction (CD) were associated with the 66HDPE group. Separate analysis of MD and CD is necessary because blown film is not isotropic: tube inflation and film take-up induce orientation during extrusion.

These conclusions are consistent with previous studies on three-layer PE films containing recycled polyethylene, which showed that increasing the recycled content to approximately 54% may allow the required mechanical properties to be maintained, provided that the recycled raw materials are properly classified and dosed under controlled conditions [2]. This consistency is important, as it confirms that, at high recycled polyethylene contents, formula-related considerations are essential and cannot be replaced by process optimisation alone.

From a practical perspective, recycled-polyethylene content should not be considered independently of recycled-feedstock type and the complete film formula. The observed differences between the 54HDPE and 66HDPE groups therefore represent differences between the investigated formulas, rather than a separately established causal effect of increasing recycled content from the nominal level of 54% to 66%.

On this basis, it can be concluded that the material composition determines the film’s inherent capacity for deformation and load transfer, RAD governs the intensity of local deformation, and the embossing pattern modifies the distribution of these deformations.

- The context of the embossing process

The results do not support the simple conclusion that every geometric embossing pattern strengthens the film. A statistically significant increase in tensile strength relative to the minimum tensile strength of the corresponding non-embossed film was observed only for selected combinations: in the machine direction (σ_r_ MD) for the 54HDPE group embossed with patterns E2 and E3, and in the cross direction (σ_r_ CD) for the 66HDPE group, likewise embossed with patterns E2 and E3. For relative elongation, ε_r_ in both machine (MD) and cross direction (CD), no statistically significant improvement was observed compared with the non-embossed film.

One of the most valuable aspects of the study is the evaluation of embossing at industrial film line speeds. Within the range investigated, film line speed had no statistically significant effect on the strength results. This finding is of practical importance, as it suggests that, within the processing range investigated, the process can be carried out without reducing line throughput solely to improve mechanical strength.

Analysis of the test results indicated that the material composition of the three-layer film and the embossing roller axis distance (RAD) were the dominant factors. It was found that, even at a high recycled polyethylene content—an important consideration in view of the project’s principal practical objective—an improvement in the film’s mechanical strength can be achieved while maintaining the expected high throughput of the manufacturing process. No statistically significant effect of film line speed on the strength properties investigated was observed. This made it possible to reduce the film–roller contact time while still ensuring permanent mechanical embossing of the film, without impairing its strength properties and, with an appropriate combination of film composition, embossing pattern, and embossing conditions, even improving them.

In contrast to film line speed, the embossing roller axis distance (RAD) was a significant factor. The most favourable results were generally obtained at low and intermediate RAD settings, whereas any further increase the distance between the axes of the embossing rollers did not improve the mechanical properties. From a mechanical perspective, RAD can be regarded as a practical parameter controlling the contact intensity and embossing depth. This is one of the key technological conclusions with implications for further process development.

The main conclusions are as follows:

1. Embossing is not a universally applicable method of strengthening three-layer PE films containing recycled polyethylene. A statistically significant increase in tensile strength relative to the minimum tensile strength of the corresponding non-embossed film was confirmed only for selected combinations of material group, embossing pattern, and test direction.
2. Within the six investigated formulas, material group was the dominant factor differentiating tensile strength; the design does not separate the effect of recycled content from recycled-polyethylene type and other compositional differences. The most favourable MD tensile-strength results were obtained for the 54HDPE group, whereas the 66HDPE group exhibited the most favourable CD results.
3. The effect of the embossing pattern depended on the other factors investigated. The E2 and E3 patterns produced favourable results in specific configurations but did not act as independent reinforcing factors irrespective of the material composition.
4. Within the industrial processing range investigated, the film line speed had no statistically significant effect on the tensile strength results. This is advantageous from a processing perspective, as it suggests that industrial production speeds can be used without compromising mechanical performance.
5. The statistically significant effect of the embossing roller axis distance (RAD) indicates that embossing intensity is a critical process parameter. The most favourable results were obtained at low and intermediate RAD settings, whereas excessive increase in the distance between the axes of the embossing rollers produced no further improvement in the mechanical properties.
6. Tensile strength of in the machine direction (MD) and cross direction (CD) must be analysed separately because blown films exhibit structural and mechanical anisotropy. Results obtained in one direction should not be directly extrapolated to the other.
7. Exploratory evaluation of factor interactions underscores the need for an integrated optimization approach, in which material formula, embossing pattern geometry, and roller axis distance (RAD) are evaluated in combination rather than as strictly isolated variables.
8. The configurations associated with favourable measured static tensile-property results combined a predominantly HDPE recyclate fraction with the E2 or E3 embossing pattern and moderate embossing intensity.
9. Industrial processing was demonstrated for the six investigated formulas at nominal recycled-polyethylene contents of 54% and 66%, corresponding to average calculated mass fractions of 54.2% and 65.8% across the three-layer film. At both nominal levels, acceptable tensile-strength and elongation values in MD and CD were obtained for selected formulas under appropriate processing conditions; this does not imply acceptance of every embossing configuration tested. These findings apply to the investigated formulas and conditions only. Industrial constraints limited sampling at additional recycled-content levels, and the results do not establish processability at all contents above 54%, at intermediate contents, or at higher contents. Results from outside the present six-formula analysis are not used to extend these conclusions.

## Figures and Tables

**Table 1:** Three-layer film formulas selected for the investigation of the effect of embossing on mechanical properties.

Group Name	Formula Code	Types of Polyethylene Used	Total Proportion of Recycled Polyethylene in Three-Layer Film [%]
54LDPE	F004	virgin: LD-PE, recyclate: LD-PE	54.2
	F032		
54HDPE	F046	virgin: LD-PE, recyclate: LD-PE	
	F047		
66HDPE	F045	virgin: LD-PE, recyclate: LD-PE + HD-PE	65.8
	F050		

**Table 2:** Mean static tensile strength and relative elongation of non-embossed three-layer films.

Formula Code	σ_r_ (MD) [MPa]	σ_r_ (CD) [MPa]	ε_r_ (MD) [%]	ε_r_ (CD) [%]
F004	21.86	20.62	562.24	629.44
F032	24.74	22.85	583.28	715.08
F045	24.63	24.48	561.13	668.29
F046	21.78	22.57	506.48	624.74
F047	26.72	22.34	589.36	673.38
F050	23.73	18.08	478.30	597.66

**Table 3:** Summary of the mean values of static tensile strength and relative elongation for a group of specimens prepared from a three-layer film produced according to a single formula and embossed with a single embossing pattern at different film line speeds and the embossing roller axis distance settings (only for formula F004, embossing pattern E2).

RAD	Film Feed Speed [m/min]	σ_r_ (MD) [MPa]	σ_r_ (CD) [MPa]	ε_r_ (MD) [%]	ε_r_ (CD) [%]
0	150	17.62	13.33	158.54	10.64
1	150	29.99	23.47	438.71	580.37
2	150	25.28	23.67	375.20	578.78
3	150	32.14	25.02	456.28	594.07
4	150	32.48	23.48	446.67	572.56
5	150	25.56	19.03	370.21	488.98
6	150	24.93	11.91	396.26	95.64
8	150	20.41	13.09	292.68	70.23
0	165	17.59	13.07	176.50	11.005
1	165	27.85	22.32	421.01	572.66
2	165	27.16	23.18	418.92	580.75
3	165	28.42	20.86	441.45	558.73
4	165	24.10	20.73	380.39	547.43
5	165	25.14	20.33	371.57	517.26
6	165	23.92	15.23	374.03	349.07
8	165	22.28	12.99	342.03	158.99
0	180	18.99	21.06	277.20	517.49
1	180	30.18	22.55	444.59	593.00
2	180	25.09	20.55	371.49	541.54
3	180	31.86	23.95	455.24	587.49
4	180	28.91	19.44	438.25	500.35
5	180	27.42	21.06	398.67	527.58
6	180	24.73	19.34	411.14	478.75
8	180	22.10	15.71	359.57	318.65
0	195	26.00	22.69	399.38	586.06
1	195	24.38	19.37	357.35	520.53
2	195	29.82	24.39	452.23	594.83
3	195	20.96	24.91	256.023	586.57
4	195	28.41	21.20	414.78	532.55
5	195	25.06	24.70	385.40	565.53
6	195	22.11	17.47	305.74	402.33
8	195	25.25	19.43	356.72	455.20

**Table 4:** Statistical test results for cases showing a significant increase in tensile strength in the machine direction, σ_r_ MD, relative to the non-embossed film.

Measurement Direction	Material Group	Embossing Pattern	Number of Observations	Wilcoxon Test *p*-Value (Holm-Adjusted)	Wilcoxon Test Effect Size	Sign Test *p*-Value (Holm-Adjusted)	Sign Test Effect Size
σ_r_ MD	54HDPE	E2	63	1.50⋅10^−7^	0.683	5.02⋅10^−7^	0.651
σ_r_ MD	54HDPE	E3	48	4.82⋅10^−8^	0.736	8.26⋅10^−6^	0.667

**Table 5:** Statistical test results for cases showing a significant increase in tensile strength in the cross direction (σ_r_ CD) relative to the non-embossed film.

Measurement Direction	Material Group	Embossing Pattern	Number of Observations	Wilcoxon Test *p*-Value (Holm-Adjusted)	Wilcoxon Test Effect Size	Sign Test *p*-Value (Holm-Adjusted)	Sign Test Effect Size
σ_r_ CD	66HDPE	E2	64	0.0051	0.386	0.0092	0.375
σ_r_ CD	66HDPE	E3	50	0.0002	0.557	0.0078	0.440

**Table 6:** Results of the statistical tests comparing the mean tensile strength in the machine direction (σ_r_ MD) of embossed films from the 54HDPE group with that of non-embossed films, taking into account the embossing roller axis distance (RAD).

Measurement Direction	Material Group	Embossing Pattern	RAD	Number of Observations	Wilcoxon Test *p*-Value (Holm-Adjusted)	Wilcoxon Test Effect Size	Sign Test *p*-Value (Holm-Adjusted)	Sign Test Effect Size
σ_r_ MD	54HDPE	E2	1	8	0.1875	0.891	0.1875	1
σ_r_ MD	54HDPE	E2	2	8	0.1875	0.891	0.1875	1
σ_r_ MD	54HDPE	E2	3	8	0.1875	0.891	0.1875	1
σ_r_ MD	54HDPE	E2	4	8	0.1875	0.891	0.1875	1
σ_r_ MD	54HDPE	E2	5	8	0.3437	0.841	1	0.750
σ_r_ MD	54HDPE	E2	6	8	1	0.693	1	0.693
σ_r_ MD	54HDPE	E3	0	6	0.6719	0.898	0.6875	0.898
σ_r_ MD	54HDPE	E3	1	6	0.6719	0.898	0.6875	0.898
σ_r_ MD	54HDPE	E3	2	6	0.6719	0.898	0.6875	0.898
σ_r_ MD	54HDPE	E3	3	6	0.6719	0.898	0.6875	0.898
σ_r_ MD	54HDPE	E3	4	6	0.6719	0.898	0.6875	0.898

**Table 7:** Results of the statistical tests comparing the tensile strength in the cross direction (σ_r_ CD) of embossed films from the 66HDPE group with that of non-embossed films, taking into account the embossing roller axis distance (RAD).

Measurement Direction	Material Group	Embossing Pattern	RAD	Number of Observations	Wilcoxon Test *p*-Value (Holm-Adjusted)	Wilcoxon Test Effect Size	Sign Test *p*-Value (Holm-Adjusted)	Sign Test Effect Size
σ_r_ CD	66HDPE	E2	1	8	1	0.643	1	0.750
σ_r_ CD	66HDPE	E2	2	8	0.551	0.792	1	0.750
σ_r_ CD	66HDPE	E2	3	8	0.375	0.841	1	0.750
σ_r_ CD	66HDPE	E3	0	6	1	0.812	1	0.667
σ_r_ CD	66HDPE	E3	1	6	1	0.812	1	0.667
σ_r_ CD	66HDPE	E3	3	6	1	0.812	1	0.667
σ_r_ CD	66HDPE	E3	4	6	0.719	0.898	0.75	1

**Table 8:** Results of the comparisons among the recycled polyethylene groups without accounting for additional process variables.

Parameter	Global Test Result	Statistically Significant Pairwise Comparisons	Group(s) with More Favourable Results
σ_r_ MD	*p* = 3.096·10^−13^	54LDPE–54HDPE, *p* < 0.0001; 54LDPE–66HDPE, *p* = 0.000134; 54HDPE–66HDPE, *p* = 0.000004	54HDPE
ε_r_ MD	*p* = 2.431·10^−10^	54LDPE–66HDPE, *p* < 0.0001; 54HDPE–66HDPE, *p* < 0.0001	54LDPE, 54HDPE
σ_r_ CD	*p* = 0.000217	54LDPE–66HDPE, *p* = 0.000139; 54HDPE–66HDPE, *p* = 0.029020	66HDPE
ε_r_ CD	*p* = 2.032·10^−9^	54LDPE–54HDPE, *p* < 0.0001; 54HDPE–66HDPE, *p* < 0.0001	54LDPE, 66HDPE

**Table 9:** Summary of multifactorial non-parametric statistical model for recycled polyethylene content and embossing pattern.

Response Variable	Fixed Main Factors	Specified Interaction Terms
σ_r_ MD	Recycled Polyethylene Content, Embossing Pattern	Recycled Polyethylene Content × Embossing Pattern (54LDPE, 54HDPE, 66HDPE) × (E2,E3)
ε_r_ MD		
σ_r_ CD		
ε_r_ CD		

**Table 10:** Results of the Scheirer–Ray–Hare test for the effects of recycled polyethylene content and embossing pattern and ART ANOVA for their interaction.

Parameter	Effect of Recycled Polyethylene Content	Effect of Embossing Pattern	Recycled Polyethylene Content × Embossing Pattern Interaction Effect	Most Favourable Results
σ_r_ MD	*p* < 0.000001	*p* = 0.006056	*p* = 0.036391	54HDPE; E2 and E3; especially 54HDPE E2 and 54HDPE E3
ε_r_ MD	*p* < 0.000001	*p* = 0.00000173	*p* = 0.002204	54LDPE and 54HDPE; favourable results for selected combinations with the E3 embossing pattern
σ_r_ CD	*p* = 0.000086	*p* = 0.165284	*p* = 0.067703	66HDPE; E2 and E3
ε_r_ CD	*p* < 0.000001	*p* < 0.000001	*p* = 0.026314	54LDPE and 66HDPE; favourable results for selected combinations with the E3 embossing pattern

**Table 11:** Summary of multifactorial non-parametric statistical model for regranulate content and film line speed.

Response Variable	Fixed Main Factors	Specified Interaction Terms
σ_r_ MD	Recycled Polyethylene Content, Film line speed	Recycled polyethylene Content × Film line speed (54LDPE, 54HDPE, 66HDPE) × (150,165,180,195)
ε_r_ MD		
σ_r_ CD		
ε_r_ CD		

**Table 12:** Results of the Scheirer–Ray–Hare test for the effects of recycled polyethylene content and film line speed and ART ANOVA for their interaction.

Parameter	Effect of Recycled Polyethylene Content	Effect of Film Line Speed	Recycled Polyethylene Content × Film Line Speed Interaction	Group(s) with More Favourable Results
σ_r_ MD	*p* < 0.00001	*p* = 0.82623	*p* = 0.43682	54HDPE
ε_r_ MD	*p* < 0.00001	*p* = 0.64480	*p* = 0.79276	54LDPE and 54HDPE
σ_r_ CD	*p* = 0.00008	*p* = 0.87672	*p* = 0.99467	66HDPE
ε_r_ CD	*p* < 0.00001	*p* = 0.11783	*p* = 0.99952	54LDPE and 66HDPE

**Table 13:** Summary of multifactorial non-parametric statistical model for recycled polyethylene content and embossing roller axis distance.

Response Variable	Fixed Main Factors	Specified Interaction Terms
σr MD	Recycled Polyethylene Content, Embossing roller axis distance	Recycled Polyethylene Content × Embossing roller axis distance (54LDPE, 54HDPE, 66HDPE) × (0,1,2,3,4,5,6,8)
εr MD		
σr CD		
εr CD		

**Table 14:** Results of the Scheirer–Ray–Hare test for the effects of recycled polyethylene content and embossing roller axis distance and ART ANOVA for their interaction.

Parameter	Effect of Recycled Polyethylene Content	Effect of RAD	Recycled Polyethylene Content × RAD Interaction	Group(s) with More Favourable Results	Favourable RAD Levels
σ_r_ MD	*p* < 0.00001	*p* < 0.00001	*p* = 0.92915	54HDPE	0, 1, 2, 3, 4, 5
ε_r_ MD	*p* < 0.00001	*p* < 0.00001	*p* = 0.94469	54LDPE i 54HDPE	0, 1, 2, 3, 4
σ_r_ CD	*p* = 0.00044	*p* < 0.00001	*p* = 0.75968	66HDPE	0, 1, 2, 3, 4
ε_r_ CD	*p* < 0.00001	*p* < 0.00001	*p* = 0.60569	54LDPE i 66HDPE	1, 2, 3

**Table 15:** Combinations of material group (recycled polyethylene content), embossing pattern, and embossing roller axis distance for which a statistically significant improvement relative to the corresponding non-embossed film was demonstrated.

Parameter	Material Group	Embossing Pattern	RAD	Conclusion
σ_r_ MD	54HDPE	E2	1, 2, 3, 4, 5	Large effect sizes suggest a potential improvement in tensile strength relative to the non-embossed film.
σ_r_ MD	54HDPE	E3	0, 1, 2, 3, 4	Large effect sizes suggest a potential improvement in tensile strength relative to the non-embossed film.
σ_r_ CD	66HDPE	E2	1, 2, 3	Large effect sizes suggest a potential improvement in tensile strength relative to the non-embossed film.
σ_r_ CD	66HDPE	E3	4	Large effect sizes suggest a potential improvement in tensile strength relative to the non-embossed film.
ε_r_ MD	—	—	—	No significant improvement
ε_r_ CD	—	—	—	No significant improvement

**Table 16:** Summary of the levels of the grouping factors associated with more favourable mechanical properties of embossed films.

Parameter	Recycled Polyethylene Content	Embossing Pattern	Film Feed Speed	RAD
σ_r_ MD	54HDPE	E2, E3	no significant impact	0, 1, 2, 3, 4, 5
ε_r_ MD	54LDPE, 54HDPE	E3—favourable results for selected combinations	no significant impact	0, 1, 2, 3, 4
σ_r_ CD	66HDPE	No statistically significant overall effect of the embossing pattern	no significant impact	0, 1, 2, 3, 4
ε_r_ CD	54LDPE, 66HDPE	E3—favourable results for selected combinations	no significant impact	1, 2, 3

## Data Availability

The original contributions presented in this study are included in the article. Further inquiries can be directed to the corresponding author.
